# Führen Online-Befragungen zu anderen Ergebnissen als persönliche Interviews? Eine Schätzung von Moduseffekten am Beispiel eines Mixed-Mode Surveys

**DOI:** 10.1007/s11614-023-00532-4

**Published:** 2023-06-05

**Authors:** Bernd Liedl, Nadia Steiber

**Affiliations:** Institut für Soziologie, Universität Wien, Rooseveltplatz 2, 1090 Wien, Österreich

**Keywords:** Befragungsmodus, Moduseffekte, Online-Befragung, CAWI, CATI, Survey mode, Mode effects, Online survey, CAWI, CATI

## Abstract

Der zunehmende Einsatz von Online-Befragungen (CAWI) ist ein Trend, der sich im Zuge der COVID-19 Pandemie weiter verstärkte. Der Umstieg von persönlichen Interviews auf Online-Befragungen wirft die Frage nach Moduseffekten auf. Unterscheiden sich die Ergebnisse von Online-Befragungen von jenen auf Basis von Telefoninterviews? Die vorliegende Studie untersucht diese Frage unter Verwendung der Daten aus dem AKCOVID Panel Survey, der ein Mixed-Mode Design aufweist und im ersten Jahr der COVID-19 Pandemie durchgeführt wurde. Die Ergebnisse von Regressionsanalysen für 46 verschiedene Variablen, die für die Zusammensetzung der CAWI- und CATI-Stichproben kontrollieren, zeigen, dass Moduseffekte auf bestimmte Themenbereiche eingegrenzt werden können. Für manche Themen werden signifikante und substantiell bedeutsame Moduseffekte geschätzt (soziales Vertrauen, Zukunftssorgen, finanzielle Problemlagen und Gesundheit). Telefonisch Befragte antworten stärker sozial erwünscht, bringen Sorgen weniger stark zum Ausdruck, schätzen ihre finanzielle Lage, ihren sozialen Status und ihre Gesundheit besser ein, und sie geben an, anderen Menschen mehr zu vertrauen und prosozialer eingestellt zu sein als Online-Befragte. Kaum von Moduseffekten betroffen zeigen sich ‚faktische‘ Fragen nach krisenbedingten Veränderungen des Einkommens ohne Bezug zur finanziellen Lage, Fragen zu Arbeitsbedingungen, familiären Beziehungen oder vielen politischen Einstellungen. Der Beitrag schließt, dass Ergebnisse persönlicher Befragungen aus Zeiten vor der Pandemie oft nicht direkt mit aktuellen Ergebnissen auf Basis von Online-Erhebungen verglichen werden können und weist auf die Bedeutung heterogener Moduseffekte hin, deren systematische Untersuchung und Berücksichtigung als Forschungslücke identifiziert wird.

**Zusatzmaterial online:**

Zusätzliche Informationen sind in der Online-Version dieses Artikels (10.1007/s11614-023-00532-4) enthalten.

## Abstract (en)

The increasing use of online surveys (CAWI) is a trend that became more pronounced in the wake of the COVID-19 pandemic. The switch from face-to-face interviews to online surveys raises the question of mode effects. Do the results of online surveys differ from those based on telephone interviews? This study examines this question using data from the AKCOVID Panel Survey, which has a mixed-mode design and was conducted in the first year of the COVID-19 pandemic. The results of regression analyses for 46 different variables controlling for the sample characteristics of CAWI and CATI show that mode effects are topic-specific. For some topics, significant and substantial mode effects are estimated (social trust, worries about the future, financial problems and health). When people are interviewed by telephone, they answer more socially desirable, express worries less strongly, assess their financial situation, social status and health better, report more trust in other people, and present themselves as more prosocial than in CAWI interviews. Not or less affected by mode effects are ’factual’ questions about crisis-related changes in income without reference to one’s financial situation, questions about working conditions, family relations, or a range of political attitudes. The paper concludes that results of interviewer-led surveys from pre-pandemic times can often not be directly compared with current results based on online surveys and points to the importance of *heterogeneous* mode effects that are hitherto under-researched.

## Einleitung

Die COVID-19 Pandemie hat den langjährigen Trend, sozialwissenschaftliche Befragungen zunehmend im Onlinemodus durchzuführen, noch weiter verstärkt. Einerseits wurden in der Pandemie Face-to-Face Befragungen im Sinne von Kontaktbeschränkungen vermieden, andererseits wurde versucht, durch Online-Befragungen zeitnah Erkenntnisse über Entwicklungen während der Pandemie zu erhalten. Der Onlinemodus wird nicht zuletzt aus Kostengründen immer häufiger eingesetzt und mit anderen Befragungsmodi kombiniert. Durch *Mixed-Mode Designs* wird versucht, Selektionseffekte unterschiedlicher Befragungsmodi auszugleichen. Im Onlinemodus fällt jedoch die direkte Interaktion zwischen Interviewer*innen und Befragten weg, womit sich die methodische Frage stellt, ob und wie sich der Befragungsmodus auf das Antwortverhalten auswirkt. Unterscheiden sich die Ergebnisse von Online-Befragungen von jenen, die von Interviewer*innen durchgeführt werden?

Die Abwesenheit eine*r Interviewer*in bei Online-Befragungen kann bei Fragen, die dazu verleiten sozial erwünschte Antworten zu geben, gewährleisten, dass Befragte ehrlicher antworten; umgekehrt kann das selbständige Ausfüllen von Online-Umfragen aber auch dazu führen, dass Fragen nicht richtig verstanden oder mit minimalem kognitiven Aufwand beantwortet werden (z. B. schnelles ‚Durchklicken‘, Antworttendenzen zur Mitte oder zu den Rändern von Antwortskalen, cf. Mühlböck et al. 2017). Moduseffekte dieser Art, welche über die unterschiedliche Zusammensetzung von Stichproben je nach Modus hinausgehen, sind Gegenstand der vorliegenden Untersuchung. Wir vergleichen die beiden Befragungsmodi des AKCOVID Panel Surveys (Steiber 2021): computerunterstützte Telefoninterviews (CATI) und computerunterstützte Webinterviews (CAWI).

Nachdem wir in den Abschn. 2 und 3 den Stand der Forschung und das Design der Studie erörtern, legen wir in den Abschn. 4.1–4.3 die Effekte der beiden Befragungsmodi auf das Antwortverhalten dar. In Abschn. 4.4 illustrieren wir anhand eines Beispiels, dass Befragungen auf Basis unterschiedlicher Modi nicht vergleichbar sind, und diskutieren die Bedeutung von heterogenen Moduseffekten in verschiedenen Bevölkerungsgruppen.

## Stand der Forschung: Moduseffekte in der Umfrageforschung

Die Befundlage zu Moduseffekten ist durchwachsen. Manche Studien kommen zum Schluss, dass Befragungen auf Basis unterschiedlicher Befragungsmodi niemals zu vergleichbaren Ergebnissen führen, weil sich der Modus der Befragung signifikant auf das Antwortverhalten auswirkt – ganz unabhängig vom Thema der Untersuchung, der Art und der ‚normativen Aufladung‘ der Fragestellung (Klausch et al. 2013). Andere Studienautor*innen vertreten die Ansicht, dass CATI und CAWI meist doch sehr ähnliche Ergebnisse liefern und nur geringe Unterschiede im Antwortverhalten festgestellt werden können (Vannieuwenhuyze und Revilla 2013, S. 164; Ansolabehere und Schaffner 2014). Eine dritte Gruppe von Studien zeigt, dass Moduseffekte unter bestimmten Umständen und bei bestimmten Gruppen stärker zu Tage treten und zielt darauf ab, genuine *Messeffekte* von *Selektionseffekten* zu unterscheiden (Schouten et al. 2013; Vannieuwenhuyze und Loosveldt 2013).

### Selektionseffekte

Vergleicht man die Ergebnisse von Online-Befragungen mit jenen von persönlichen Interviews, können etwaige Unterschiede im Antwortverhalten teils darauf zurückgeführt werden, dass sich die Stichproben der beiden Befragungsmodi voneinander unterscheiden können *(Selektionseffekte).* In vielen *Mixed-Mode Designs* wird eine Online-Stichprobe um eine Telefonstichprobe ergänzt, in der Hoffnung, die Repräsentativität der Gesamtstichprobe auf diesem Weg zu optimieren. Dabei werden meist spezifische Gruppen – wie etwa ältere Personen – bei den Telefoninterviews überrepräsentiert, weil man davon ausgeht, dass diese mittels Onlinebefragung weniger gut abgedeckt werden können. In manchen Studien gibt es gar eine strikte Trennung zwischen den Modi in Bezug auf die befragten Altersgruppen (z. B. der IHS COVID-19 Survey, cf. Grand 2021). Doch auch wenn unterschiedliche Zusammensetzungen der Telefon- und Online-Stichproben nicht intendiert sind, kann nicht ausgeschlossen werden, dass sich die beiden Gruppen anhand von Eigenschaften voneinander unterscheiden, die sich auf das Antwortverhalten auswirken (z. B. Lesekompetenz, digitale Kompetenz, politisches Interesse).

Erfolgt bei CATI oft eine einfache *Zufallsauswahl* der Befragten (z. B. RDD-Random Digit Dialing), kommen bei Online-Befragungen in der Praxis v. a. aus Kostengründen häufig große *Online-Access Panels*^1^ zur Anwendung (z. B. Austrian Corona Panel Project (Kittel et al. 2021), Values in Crisis Austria (Aschauer et al. 2020) sowie auch der CAWI-Teil des AKCOVID Panel Surveys (Steiber 2021)). Für Befragungen auf Basis von *Online-Access Panels* werden Respondent*innen nicht auf Basis einer Zufallsauswahl rekrutiert, sondern sind bereits in einem Pool an Personen, die sich studienunabhängig bereiterklärt haben, an Online-Umfragen teilzunehmen. Aus diesem Pool werden Personen ohne Onlinezugang klarerweise ausgeschlossen, während webaffine Personen mit überdurchschnittlich hoher Lesekompetenz bzw. stärkerem politischen Interesse überrepräsentiert sind (Prandner 2022, S. 337). Es besteht das zentrale methodische Problem der Selbstselektion in den Pool der möglichen Befragten. Dies kann die Repräsentativität von Teilstichproben aus diesem Pool gefährden.

Über reine *Selektionseffekte* hinausgehend – bzw. unter Kontrolle der Zusammensetzung der Stichproben unterschiedlicher Befragungsmodi – können Unterschiede im Antwortverhalten je nach Befragungsmodus auch durch genuine *Messeffekte* bedingt sein. Diese *Messeffekte* stehen im Zentrum der gegenständlichen Analyse.

### Effekte auf die Item-Non-Response

*Messeffekte* können durch verschiedene Muster der Antwortverweigerung (*Non-Response*) entstehen oder durch unterschiedliche Antwortmuster bedingt sein (De Leeuw und Hox 2015, S. 22–34). Befragte tendieren am Telefon beispielsweise eher dazu, die letzte Antwortoption zu wählen, während bei Webinterviews eher die erste gewählt wird, v. a. dann, wenn nur die Endpunkte einer Skala beschriftet sind. Weiters wird berichtet, dass die Option „weiß nicht“ bei CAWI signifikant häufiger gewählt wird als dies bei CATI der Fall ist (De Leeuw und Hox 2015; Bowyer und Rogowski 2017). Das kann bei einem Teil der Befragten ein Problem sein, wenn diese die Befragung mit einem minimalen zeitlichen und kognitiven Aufwand beenden wollen (Callegaro et al. 2015, S. 102). Zudem kann die soziale Situation einer persönlichen Befragung dazu verleiten, Antworten zu geben, auch wenn Befragte keine eindeutige Antwort auf die Frage haben (Atteslander 2008, S. 110–111; Peytchev und Hill 2010). Dieser Argumentation folgend sollten die Non-Response-Raten bei CAWI höher ausfallen als bei CATI (Hypothese 1a).

Umgekehrt wurde argumentiert, dass die Item-Non-Response bei persönlichen Interviews höher ausfallen könnte als bei Online-Befragungen und zwar bei sensitiven Fragestellungen. Manche Fragen werden als zu privat oder aufdringlich empfunden, wie etwa Fragen zu Einkommen oder Partnerschaft und werden deswegen weniger gerne beantwortet (Tourangeau und Yan 2007). Die Rate der Antwortverweigerung auf sensitive Fragen kann dabei bei persönlichen Interviews besonders hoch ausfallen, während die Abwesenheit von Interviewer*innen bei CAWI dazu führen kann, dass mehr Befragte sensitive Fragen beantworten und damit die Rate der Antwortverweigerung niedriger ausfällt (Joinson et al. 2007; Kreuter et al. 2008). Dieser Argumentation folgend sollten die Non-Response-Raten bei sensitiven Fragen generell höher ausfallen und man würde hier stärkere Moduseffekte zugunsten geringerer Non-Response bei CAWI als bei CATI erwarten (Hypothese 1b). Im AKCOVID Panel Survey können Fragen in den Frageblöcken *Soziale Beziehungen* und *Finanzielle Lage* als sensitive private Fragen identifiziert werden, bei denen wir im CAWI-Modus weniger Non-Response erwarten.

### Soziale Erwünschtheit

*Messeffekte* können auch durch sozial erwünschtes Antwortverhalten bedingt sein. Befragte tendieren dazu, in ihren Antworten sozial normierten Erwartungen entsprechen zu wollen (Tourangeau und Smith 1996; Joinson et al. 2007; Kreuter et al. 2008; Malakhoff und Jans 2011). Sozial erwünschtes Antwortverhalten tritt dann verstärkt auf, wenn es um Einstellungen und Verhaltensweisen geht, die stärker durch soziale Normen geregelt werden. Ein gutes Beispiel für einen Themenbereich, in dem soziale Erwünschtheit das Antwortverhalten beeinflusst, sind Fragen zu kriminellem Verhalten. Diese werden häufig sozial erwünscht beantwortet, da hier explizit ein Normbruch thematisiert wird. Es gibt aber auch bei scheinbar weniger sensiblen Fragen die Tendenz, sich selbst positiv darzustellen. Beispielsweise werden Fragen zum persönlichen Wohlbefinden häufig den sozialen Erwartungen entsprechend beantwortet (Reinecke 1991, S. 105) – klassische Antwort: „Mir geht’s gut!“.

Während es bei zu aufdringlichen Fragen zu privaten Lebensbereichen wahrscheinlich eher als legitim empfunden wird, die Antwort zu verweigern (Non-Response, siehe Abschn. 2.2), kann die Verweigerung einer Antwort auch ein Gefühl der Selbstentlarvung hervorrufen. Beispielsweise könnten Befragte die Sorge haben, dass die Nichtbeantwortung von Fragen zum Thema Kriminalität implizit als Indikator für ein bestimmtes Verhalten gewertet werden könnte. Ein anderes Beispiel sind Fragen zum Gesundheitszustand. Die Antwort wird in manchen Fällen daher in Richtung sozialer Erwünschtheit modifiziert werden (Tourangeau und Yan 2007).

Kommt es dabei auf den Befragungsmodus an? Eine Reihe von Studien kommt zum Schluss, dass der Effekt der sozialen Erwünschtheit bei CATI stärker ausgeprägt ist als bei CAWI (Holbrook und Krosnick 2010; Engel et al. 2015; Bowyer und Rogowski 2017). Während durch die akustische Anwesenheit von Interviewer*innen bei telefonischen Befragungen die sozialen Normen in Erinnerung gerufen werden, wird in der völlig anonymen Situation der Web-Befragung eher ‚ehrlich‘ geantwortet. Damit wären bei sensitiven Fragen stärkere Messeffekte zu erwarten. Im AKCOVID Panel Survey können Fragen in den Frageblöcken *Finanzielle Lage, Sorgen, Kinder, Gesundheit, Einstellungen *und* Soziale Beziehungen* als jene mit Potenzial für sozial erwünschtes Antwortverhalten eingestuft werden. In diesen Themenblöcken erwarten wir mithin im CAWI-Modus weniger in Richtung sozialer Erwünschtheit verzerrte Antworten, während im CATI-Modus Antworten stärker in Richtung einer sozial erwünschten *guten* finanziellen Lage und Gesundheit, *intakten* sozialen Beziehungen und *sozialeren* Einstellungen erwartet werden (Hypothese 2).

## Design der vorliegenden Studie: Vergleich CAWI mit CATI

Ziel der vorliegenden Untersuchung ist die Schätzung von Moduseffekten im AKCOVID Panel Survey (Steiber 2021) durch die Analyse unterschiedlichen Antwortverhaltens bei Telefonbefragungen (CATI) im Vergleich zu Online-Befragungen (CAWI). Dazu vergleichen wir die beiden Befragungsmodi des AKCOVID Panel Surveys. Im Rahmen der AKCOVID Studie wurden im Juni 2020 zwei Tausend in Österreich wohnhafte Personen im Alter zwischen 20 und 64 Jahren befragt: 80 % Online ohne Interviewer*in und 20 % mittels Interviewer*in per Telefon, beides computerunterstützt auf Basis eines identen Fragebogens. Die Anteile einzelner Bevölkerungsgruppen (definiert nach Alter, Bildung, Geschlecht und Bundesland) in der CATI Stichprobe wurden so gewählt, dass die Gesamtstichprobe der Befragung (CAWI plus CATI) der Struktur der Gesamtbevölkerung nach diesen Kriterien entsprach. Im Jänner 2021 wurden rund 70 % der in der Ersterhebung Teilnehmenden ein zweites Mal befragt.

Ziel dieser Studie ist, herauszufinden, ob für alle Items der Befragung ähnliche Moduseffekte gefunden werden können oder ob sich modusbedingte Verzerrungen auf bestimmte Item-Designs, Themen oder Bevölkerungsgruppen beschränken. Um diesen Fragen nachzugehen, vergleichen wir für 46 Variablen das Ausmaß der *Non-Respons*e („weiß nicht“ Antworten oder „keine Angabe“ bzw. „Kann ich nicht sagen“) und das *Antwortverhalten* (Mittel- und Anteilswerte) innerhalb der validen Angaben zwischen CATI und CAWI.

Zum Vergleich der Non-Response zwischen den Befragungsmodi werden die Anteile nicht-valider Antworten („weiß nicht“, „keine Angabe“) und deren Vertrauensintervalle berechnet (Prüfung Hypothesen 1a und 1b). Überlappen die Vertrauensintervalle nicht, liegt ein signifikanter Unterschied in der Non-Response zwischen CAWI und CATI vor. Messeffekte werden mittels Regressionsmodellen geschätzt (Prüfung Hypothese 2). Der Befragungsmodus fungiert dabei als zentrale erklärende Variable, wobei für die Stichprobenzusammensetzungen nach Geschlecht, Alter, Bildung, Bundesland, Urbanisierungsgrad des Wohnorts, Staatsbürgerschaft und Hauptaktivität (unterteilt in: Erwerbstätigkeit, Arbeitslosigkeit, Ausbildung, Hausarbeit, Pension) kontrolliert wird. Die Ergebnisse der Analyse sind in den Tab. 1, 2 und 3 im Überblick dargestellt. Die Gesamtmodelle inklusive der Kovariaten sind im Online Supplement verfügbar. Mit dem Ziel der Vergleichbarkeit von 46 Modellen im Sinne von Effektstärken, rechnen wir im Fall von Items mit quasi-metrischem Skalenniveau^2^ lineare Modelle (OLS) und im Fall von binär kodierten Items lineare Wahrscheinlichkeitsmodelle. Items mit ordinalem Skalenniveau werden für die in Tab. 2 und 3 dargestellten Analysen dichotomisiert. Im Sinne einer Validierung der Ergebnisse, werden jedoch für alle binären und ordinalen Variablen zusätzlich binär logistische bzw. ordinale logistische Regressionen gerechnet. Diese Zusatzanalysen führten bei allen Variablen zu vergleichbaren Ergebnissen^3^. Für Details zur Kodierung der abhängigen Variablen siehe Anhang Tab. 4.

**Table 1:** 

Variable	Ausprägung	CATI (in %)	CAWI (in %)	Total (in %)
Gender	Männer	45	51	49
	Frauen	55	49	51
Alter	20–29	10	23	20
	30–39	19	24	23
	40–49	18	24	23
	50–59	29	25	26
	60–64	24	5	9
Bildung	Pflichtschule	12	17	16
	Lehre	25	36	34
	BMS	14	14	14
	AHS-Matura	8	8	8
	BHS-Matura	10	8	9
	Diplom, Univ.-Lehrgang	13	4	6
	Hochschule	18	12	14
Bundesland	Vorarlberg	6	4	4
	Tirol	7	9	9
	Salzburg	7	6	6
	Oberösterreich	16	17	17
	Kärnten	2	7	6
	Steiermark	15	14	15
	Burgenland	3	3	3
	Niederösterreich	24	18	19
	Wien	22	21	21
Stadt/Land	Land	37	31	32
	Größeres Dorf/Kleinstadt	21	21	21
	Mittelstadt	13	16	15
	Großstadt/Vorstadt	28	32	31
Staatsbürgerschaft	Nicht AUT	6	8	8
	AUT	94	92	93
Haupttätigkeit	Vollzeit	45	43	44
	Teilzeit	20	14	15
	Kurzarbeit	8	14	13
	Ausbildung	1	4	3
	Arbeitslos	5	10	9
	Karenz, Hausarbeit	3	6	6
	Pension, Arbeitsunfähig	18	8	10

**Table 2:** 

Themenbereich	Variable/Item^a^	Moduseffekte^b,c^	Effektstärke: Beta^d^	Effektstärke: Eta Quadrat^e^	Heterogene Effekte je nach …^f^
Finanzielle Lage	Subjektive Armutsgefährdung	CAWI–schwerer zurechtkommen	−0,12***	0,009 +	–
	Finanzielle Lage: Ersparnisse/Schulden	CAWI–stärkere Zustimmung	0,10***	0,014 ++	–
	Zahlungsrückstände	CAWI–stärkere Zustimmung	0,07***	0,008 +	–
	Krisenbedingte Veränderung Verdienst	–	−0,01	–	–
	Subjektiver sozialer Status (0–10)	CATI–höher (CATI–mehr Non-Response)	−0,04**	0,007 +	–
Sorgen	Finanzielle Probleme	CAWI–mehr Sorgen	0,10***	0,013 ++	–
	Gesundheitsversorgung	CAWI–mehr Sorgen	0,06**	0,005 +	Alter
	Einkommensverlust	CAWI–mehr Sorgen	0,08***	0,011 ++	–
	Jobverlust	CAWI–mehr Sorgen	0,10***	0,013 ++	–
Kinder	Überforderung durch Home Schooling	CAWI–mehr Überforderung	−0,12***	0,015 ++	–
	Sorge Lernfortschritt der Kinder	CAWI–mehr Sorgen	−0,13***	0,016 ++	–
Gesundheit	Subjektive Gesundheit	CATI–gesünder (CAWI–mehr Non-Response)	0,04**	0,005 +	Alter
	CESD-Depressionsskala	CATI–weniger depressiv	0,05***	0,01 ++	–
Einstellungen	Einkommensumverteilung	–	−0,02	–	–
	Arbeitslosenunterstützung	CATI–stärkere Zustimmung	0,06**	0,005 +	Bildung, Alter
	Armutsbekämpfung	–	0,02	–	Bildung
	Vermögensunterschiede	(CAWI–mehr non-response)	−0,02	–	Bildung
	Unterschied Arm-Reich	(CAWI–mehr non-response)	−0,00	–	Bildung
	Soziales Vertrauen	CATI–mehr Vertrauen	−0,14***	0,041 ++	Alter

Methoden der Auswertung: Bei (quasi) metrischen Variablen lineare Regression (OLS), bei ordinalen Variablen wurden diese dichotomisiert und mittels linearer Wahrscheinlichkeitsmodelle modelliert. Für detaillierte Modell inklusive Kovariaten siehe Online-Supplement

^a^Für genaues Wording der Items im Fragebogen, siehe Anhang

^b^In der Spalte werden Moduseffekte für das Antwortverhalten berichtet, wenn ein statistisch signifikanter Effekt im Regressionsmodell auftritt

^c^Bewertung der Non-Response: Überlappen die Vertrauensintervalle der % Missings nicht, wird der Modus mit mehr Missings in Klammern ausgewiesen

^d^Effektstärke laut Regressionsmodell in Form von standardisierten Koeffizienten und deren Signifikanzniveau: * *p* < 0,05 ** *p* < 0,01 *** *p* < 0,001

^e^Effektstärker laut Regressionsmodell in Form von Eta Quadrat (+ sehr kleiner Effekt ++ kleiner Effekt). Die Richtung des Effekts ist von Spalte ’Moduseffekte’ abzulesen

^f^Heterogene Effekte wurden als Interaktion des Modus mit dem genannten Merkmal identifiziert. Es werden nur heterogene Effekte ausgewiesen, die einen signifikanten Interaktionseffekt mit dem Modus zeigen und wenn auch in getrennten Modellen zumindest für eine der beiden Ausprägungen der dichotomisierten Variablen (Bildung mit Matura =1, Altersgruppe mit 1 = 55 und älter und Geschlecht mit 1 = Frau) ein signifikanter Haupteffekt vorliegt

**Table 3:** 

Themenbereich	Variable/Item^a^	Moduseffekte^b,c^	Effektstärke: Beta^d^	Effektstärke: Eta Quadrat^e^	Heterogene Effekte je nach …^f^
Soziale Beziehungen	Zeit mit Familie	–	0,00	–	–
	Zufriedenheit mit Beziehung	–	−0,01	–	–
	Zufriedenheit Aufteilung Hausarbeit	–	−0,02	–	Alter
	Konflikt in Familie	–	0,01	–	–
	Informelle Pflege vor der Pandemie	–	−0,01	–	–
	Informelle Pflege in der Pandemie	–	−0,00	–	–
Corona-Folgen Familie/Arbeit	Kinderbetreuung	–	−0,04	–	–
	Arbeitsstunden	–	−0,03	–	–
	Zeit‑, Erfolgsdruck	–	−0,03	–	–
	Home-Office	–	0,01	–	–
	Anerkennung	–	−0,03	–	–
	Autonomie	–	−0,02	–	–
	Sicherheit Job	CATI–erhöht	−0,05**	0,005 +	–
	Planung Arbeit	–	−0,00	–	–
	Überwachung im Job	–	0,00	–	–
	Persönliche Kontakte	–	0,00	–	–
	Vereinbarkeit	–	−0,02	–	–
	Infektionsrisiko am Arbeitsplatz	–	0,02	–	–
Arbeitsbedingungen	Betriebsrat vorhanden	(CAWI–mehr non-response)	0,01	–	–
	Home-Office	–	−0,01	–	–
	Aktuelle Arbeitsstunden	(CAWI–mehr non-response)	−0,01	–	Bildung
	Wissen Vorgesetzte	(CATI–mehr non-response)	0,04	–	–
	Autonomie im Job	–	0,00	–	–
	Tätigkeit Probleme lösen	–	−0,01	–	–
	Tätigkeit eintönig	–	−0,04	–	Bildung
	Tätigkeit Dinge lernen	–	0,02	–	Geschlecht
	Tätigkeit eigene Ideen	–	0,01	–	Geschlecht

Methoden der Auswertung: Bei (quasi)metrischen Variablen lineare Regression (OLS), bei ordinalen Variablen wurden diese dichotomisiert und mittels linearer Wahrscheinlichkeitsmodelle modelliert. Für detaillierte Modell inklusive Kovariaten siehe Online-Supplement

^a^Für genaues Wording der Items im Fragebogen, siehe Anhang

^b^In der Spalte werden Moduseffekte für das Antwortverhalten berichtet, wenn ein statistisch signifikanter Effekt im Regressionsmodell auftritt

^c^Bewertung der Non-Response: Überlappen die Vertrauensintervalle der % Missings nicht, wird der Modus mit mehr Missings in Klammern ausgewiesen

^d^Effektstärke laut Regressionsmodell in Form von standardisierten Koeffizienten und deren Signifikanzniveau: * *p* < 0,05 ** *p* < 0,01 *** *p* < 0,001

^e^Effektstärker laut Regressionsmodell in Form von Eta Quadrat (+ sehr kleiner Effekt ++ kleiner Effekt). Die Richtung des Effekts ist von Spalte ’Moduseffekte’ abzulesen

^f^Heterogene Effekte wurden als Interaktion des Modus mit dem genannten Merkmal identifiziert. Es werden nur heterogene Effekte ausgewiesen, die einen signifikanten Interaktionseffekt mit dem Modus zeigen und wenn auch in getrennten Modellen zumindest für eine der beiden Ausprägungen der dichotomisierten Variablen (Bildung mit Matura = 1, Altersgruppe mit 1 = 55 und älter und Geschlecht mit 1 = Frau) ein signifikanter Haupteffekt vorliegt

## Ergebnisse

### Selektionseffekt aufgrund unterschiedlicher Zusammensetzung der Stichproben

Mittelwertvergleiche geben erste Hinweise darauf, bei welchen Fragen die Befragungsmodi zu unterschiedlichen Ergebnissen führten. Beispielsweise können im Themenbereich *Arbeitsbedingungen* signifikante Unterschiede in den Mittelwerten zwischen den Modi festgestellt werden (nicht gezeigt). Diese verlieren nach Kontrolle soziodemographischer Merkmale in den Regressionsmodellen (Tab. 3) jedoch an Bedeutung – sie sind mithin lediglich auf die unterschiedliche Zusammensetzung der Stichproben (Tab. 1) der beiden Modi zurückzuführen (z. B. höheres mittleres Alter bei CATI) und können damit als reine *Selektionseffekte* identifiziert werden.

### Wenig Unterschied zwischen CATI und CAWI in der Non-Response

Beim Vergleich der *Non-Response* zeigen sich nur bei wenigen Items Moduseffekte (im Einklang mit Befunden von Mühlböck et al. 2017). Über alle 46 Variablen hinweg kann in Bezug auf das Ausmaß der *Non-Response* kein Muster festgestellt werden (siehe Tab. 2 und 3). Bei fünf Items wurden bei CAWI häufiger die Optionen „weiß nicht“, „keine Angabe“ bzw. „Kann ich nicht sagen“ gewählt als bei CATI; bei zwei Items war es genau umgekehrt. Es können auch keine Themen oder Item-Designs ausgemacht werden, bei denen eher Moduseffekte auf die Rate der *Non-Response* auftreten. Dies gilt auch für die sensitiven Fragen zur finanziellen Situation und zum Privatleben. Die Hypothesen 1a und 1b können damit nicht bestätigt werden.

### Antwortverhalten: Mehr soziale Erwünschtheit bei persönlichen Interviews (CATI)

Der *bereinigte*, für die Zusammensetzung der CATI- und CAWI-Stichproben kontrollierte, Moduseffekt wird mit Hilfe von Regressionsmodellen geschätzt. Der bereinigte *Messeffekt* ist stärker auf den Befragungsmodus *per se* zurückführbar, soweit für alle relevanten Unterschiede zwischen den beiden Stichproben kontrolliert werden konnte.^4^ Die Ergebnisse werden in Tab. 2 präsentiert. Das genaue Wording der einzelnen Items findet sich im Anhang. Bei den Themenkreisen *soziales Vertrauen, finanzielle Probleme, Sorge um Jobverlust, Gesundheit und Probleme im Zusammenhang mit den Schulschließungen *werden signifikante Moduseffekte festgestellt:

Die Befragten gaben den CATI-Interviewer*innen gegenüber eher an, dass man anderen Menschen vertrauen kann, als online (im Schnitt um rund 1,4 Punkte mehr auf der 11-teiligen Vertrauensskala). In Zusammenhang mit einer geringeren Einschätzung des *sozialen Vertrauens* bei CAWI gab es bei diesem Befragungsmodus auch signifikant weniger Zustimmung zur Aussage, dass der Staat für einen angemessenen Lebensstandard der Arbeitslosen sorgen sollte (weniger stark ausgeprägte prosoziale Haltung). Sorgen, dass die Corona-Krise zu einer Verschlechterung der eigenen *finanziellen Lage* oder zu einem *Jobverlust* führen könnte, wurden den CATI-Interviewer*innen gegenüber signifikant seltener geäußert als in CAWI. Bei CAWI wurde weiters eher angegeben als bei CATI, dass sich die *Sicherheit des Arbeitsplatzes* seit Beginn der Pandemie verringert hat. Auch werden die *finanzielle Lage des Haushalts* und der *eigene soziale Status* bei CAWI im Mittel prekärer eingeschätzt als bei CATI. Diese Ergebnisse zeigen, dass die Einschätzung der Befragten bzgl. ihrer *finanziellen Lage* (z. B. subjektive Armutsgefährdung, finanzielle Engpässe, Rechnungsverzug) und ihres *sozialen Status* im Rahmen von Online-Befragungen im Vergleich zu persönlichen Befragungen tendenziell negativer ausfällt. Nicht oder weniger von Moduseffekten betroffen zeigen sich dagegen stärker „faktische“ Fragen nach den krisenbedingten Veränderungen des Einkommens/Umsatzes ohne Bezug auf deren Konsequenzen für die eigene finanzielle Lage.

Ein ähnliches Bild ergibt sich bei der Einschätzung der eigenen *Gesundheit*: Telefonisch Befragte schätzen sich im Schnitt gesünder ein – sowohl in Bezug auf ihren allgemeinen Gesundheitsstatus als auch im Hinblick auf ihr psychisches Wohlbefinden. Dies deckt sich mit Befunden aus der Literatur zu Indikatoren der mentalen und psychosozialen Gesundheit, die in selbstadministrierten Befragungen (CAWI oder auch Papierfragebögen) signifikant schlechter bewertet werden als bei Telefoninterviews (soziale Erwünschtheit in Präsenz von Interviewer*innen, siehe Hoebel et al. 2014; Epstein Faith et al. 2001). CAWI-Befragte äußerten auch signifikant häufiger Sorgen, dass sie aufgrund der Pandemie nicht die ärztliche Versorgung bekommen, die sie brauchen (Themenbereich *Sorgen* in Tab. 2). Ein weiteres in Tab. 2 gelistetes Thema mit signifikanten Moduseffekten sind Herausforderungen im Zusammenhang mit pandemiebedingtem Distance Learning. CAWI-Befragte gaben häufiger an, dass sie sich als Eltern überfordert fühlten und sich um den Lernfortschritt ihrer Kinder sorgten.

Die Items, bei denen signifikante Moduseffekte geschätzt werden, weisen sehr unterschiedliche Designs auf (z. B. 11-teilige Antwortskalen mit beschrifteten Rändern, fünf oder sechs vollständig beschriftete Antwortoptionen) – es kann kein Zusammenhang zwischen dem Design der Items und dem Auftreten von Moduseffekten auf das Antwortverhalten festgestellt werden.

Bei der Beantwortung der in Tab. 2 gelisteten Themen ist im Rahmen von CATI ein *stärker sozial erwünschtes bzw. auch ein positiveres Antwortverhalten* zu beobachten als bei CAWI (im Einklang mit Bowyer und Rogowski 2017). Die soziale Situation des Telefoninterviews scheint auch *prosoziale Antworten* zu fördern. In anderen Themenbereichen treten dagegen kaum Moduseffekte auf. Diese sind in Tab. 3 gelistet. Ein Beispiel sind politische Einstellungen zu den Themen Einkommensumverteilung und Armutsbekämpfung (im Einklang mit den Ergebnissen von Ansolabehere und Schaffner 2014). Auch der Themenbereich familiäre Beziehungen scheint kaum sozial erwünschtes Antwortverhalten zu triggern. Weder die Fragen nach Veränderungen in der Qualität der Paarbeziehung seit Ausbruch der Pandemie (Zufriedenheit, Konflikte) noch die Fragen zur Kinderbetreuung oder zur Vereinbarkeit von Beruf und Familie werden je nach Modus unterschiedlich beantwortet. Auch faktische Fragen zum Ausmaß der informellen Pflege (Stunden pro Woche) werden modus-unabhängig ähnlich beantwortet.

Kaum von Moduseffekten betroffen zeigt sich auch der Themenbereich *Arbeitsbedingungen*. Weder die faktischen Fragen zu den wöchentlichen Arbeitsstunden, der beruflichen Tätigkeit, dem Ausmaß der Arbeitsautonomie, der Nutzung von Home-Office und dem Vorhandensein eines Betriebsrats, noch die Fragen zu den Auswirkungen der Pandemie auf die Arbeitsbedingungen (Liste der Items in diesem Themenbereich in Tab. 4 im Anhang)^5^ zeigen sich durch den Modus der Befragung beeinflusst. Einzige Ausnahme ist die Sicherheit des Arbeitsplatzes, welche im Rahmen von CAWI negativer eingeschätzt wird.

Zusammenfassend wurden mithin entsprechend Hypothese 2 in Themenfeldern, die normativ aufgeladenen sind und sozial erwünschtes Antwortverhalten hervorrufen können, stärkere Messeffekte geschätzt, während bei Fragen, die eher auf Fakten abzielten, kaum Effekte des Befragungsmodus festgestellt werden konnten.

### Stärke der Moduseffekte und heterogene Moduseffekte

Bei signifikanten Moduseffekten gilt es zwischen vernachlässigbaren und substantiell bedeutsamen Effekten zu unterscheiden. Gängige Effektstärkemaße im Rahmen von Regressionsmodellen (z. B. Eta Quadrat) deuten durchwegs auf kleine Effekte hin (Tab. 2). Dies darf jedoch nicht darüber hinwegtäuschen, dass es sich substantiell teils um bedeutsame Effekte handelt. Beispielsweise geben CATI-Befragte im Schnitt 1,4 Punkte mehr auf der 11-teiligen Skala des sozialen Vertrauens an als CAWI-Befragte^6^ (kontrolliert für die unterschiedliche Stichprobenzusammensetzung nach zentralen soziodemografischen Merkmalen). Auch die Einschätzung der finanziellen Lage des Haushalts zeigt sich stark vom Modus der Befragung beeinflusst: So geben bei den Telefoninterviews rund 40 % der Befragten an, mit dem Haushaltseinkommen bequem auszukommen, während sich dieser Anteil bei Online-Respondent*innen auf 29 % beläuft (ebenso im Rahmen der Regressionsanalyse kontrolliert für die Stichprobenzusammensetzung). Die Ergebnisse der Studie zu den teils doch maßgeblichen Effekten des Befragungsmodus legen nahe, dass es problematisch sein kann, Ergebnisse aus persönlichen Befragungen für die Zeit vor der Pandemie – beispielsweise aus dem *European Social Survey *(ESS) oder dem *Sozialen Survey Österreich* (SSÖ) – mit neueren Ergebnissen aus Online-Befragungen zu vergleichen, insbesondere wenn die CAWI-Befragten auf Basis eines *Online Access Panels* rekrutiert wurden.

**Figure 1:**
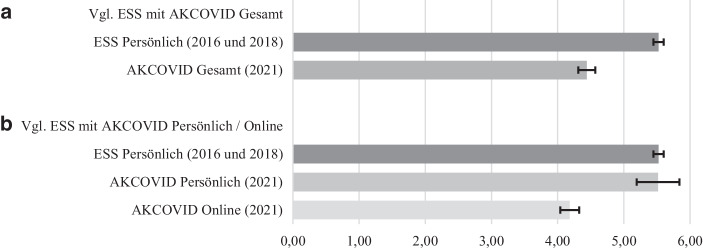

Ein durch die Autor*innen dieses Beitrags durchgeführter Vergleich von Daten aus dem European Social Survey (ESS), die in zwei Wellen zwischen 2016 und 2019 mittels persönlicher Interviews erhoben wurden (ESS 2016, 2018), und dem im Jänner 2021 erhobenen AKCOVID Panel Survey, zum mittleren *sozialen Vertrauen*, würde beispielsweise suggerieren, dass es zwischen der Erhebung der ESS Daten *vor* der Pandemie und der AKCOVID Daten im ersten Jahr der Pandemie im Jänner 2021 zu einem massiven Einbruch im Ausmaß des *sozialen Vertrauens* kam (kontrolliert für die unterschiedliche Stichprobenzusammensetzung, siehe Abb. 1a). Dieser scheinbare Trend ist jedoch zu einem großen Teil auf den Wechsel des Befragungsmodus zurückzuführen und präsentiert sich im Vergleich der mittels persönlicher Interviews erhobenen ESS-Daten mit der Telefonstichprobe aus der AKCOVID Befragung so nicht. Nur in der CAWI-Teilstichprobe des AKCOVID ist das soziale Vertrauen signifikant niedriger als im ESS (siehe Abb. 1b).

**Figure 2:**
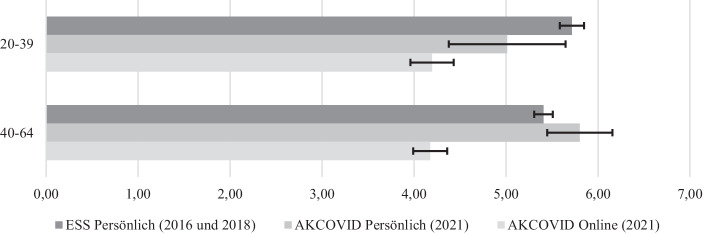

Die Analysen zum *sozialen Vertrauen* zeigen weiters, dass dieser Moduseffekt altersabhängig ist. Während es für jüngere Befragte weniger darauf ankommt, ob sie im Rahmen der AKCOVID Panelbefragung persönlich oder via Onlinesurvey zu ihrem sozialen Vertrauen befragt wurden, ist dieser Moduseffekt bei den älteren Befragten stärker ausgeprägt (Abb. 2): Während es in der jüngeren Gruppe keinen signifikanten Unterschied der geschätzten Mittelwerte zwischen CATI- und CAWI-Befragten gibt (unter 40 Jahren), signalisieren in der älteren Gruppe (40–64 Jahre) telefonisch Befragte ein signifikant höheres soziales Vertrauen.

Heterogene Moduseffekte nach Alter können in der AKCOVID-Befragung auch für die Einschätzung der eigenen Gesundheit, die Sorgen um die gesundheitliche Versorgung in der Pandemie, die Einstellung zu Arbeitslosen (Tab. 2) und die Zufriedeneheit mit der Aufteilung der Hausarbeit (Tab. 3) festgestellt werden. Heterogene Moduseffekte nach dem höchsten Bildungsabschluss der Befragten zeigen sich bei den politischen Einstellungen (Tab. 2) und den Arbeitsstunden (Tab. 3), nach dem Geschlecht der Befragten bei den Arbeitsbedingungen (Tab. 3).

## Resümee und Ausblick

Die vorliegenden Analysen des Antwortverhaltens in einem Mixed-Mode Survey legen nahe, dass Befragungen je nach Modus (CATI versus CAWI) in der Tat zu sehr unterschiedlichen Ergebnissen führen können. Zwar unterscheidet sich das Antwortverhalten kaum in Bezug auf die *Non-Response-Rate*, inhaltlich differieren die Antworten aber teils signifikant zwischen den Befragungsmodi (kontrolliert für die Stichprobenzusammensetzung nach Modus). Moduseffekte im AKCOVID Panel Survey können auf bestimmte Themenbereiche eingegrenzt werden: Vor allem bei Fragen zu finanziellen Problemlagen, der Einschätzung des eigenen sozialen Status und der eigenen Gesundheit, bei der Thematisierung von Sorgen um die Zukunft und zum eigenen sozialen Verhalten unterscheidet sich das Antwortverhalten signifikant nach Modus. Von Interviewer*innen per Telefon Befragte antworten stärker sozial erwünscht, beurteilen ihre finanzielle, soziale und gesundheitliche Lage positiver, bringen Sorgen weniger stark zum Ausdruck und geben an, anderen Menschen mehr zu vertrauen und prosozialer eingestellt zu sein als CAWI-Befragte.

Einschränkend muss angemerkt werden, dass zwei sehr unterschiedliche Befragungsmodi miteinander verglichen wurden. Die per Telefon Befragten wurden im Gegensatz zu den Online-Respondent*innen von Interviewer*innen befragt. Sie wurden darüberhinausgehend anders rekrutiert: Die CATI-Stichprobe wurde zufallsbasiert mittels Random Digit Dialing erstellt; die CAWI-Befragten dagegen aus einem bestehenden *Online-Access Panel *rekrutiert. Damit können wir strenggenommen den Effekt des persönlichen Interviews nicht von potenziellen *Selektionseffekten* isolieren. Die Regressionsanalysen kontrollieren für die unterschiedliche Stichprobenzusammensetzung der CAWI- und CATI-Befragten nach sozioökonomischen Merkmalen, ein verbleibender *Selektionsbias* auf Basis unbeobachteter Merkmale kann aber nicht ausgeschlossen werden.

Welche Schlüsse können auf Basis der Studienergebnisse für Online-Befragungen gezogen werden? Rein webbasierte Befragungen können den Vorteil bieten, dass sensitive Fragen eventuell ehrlicher beantwortet werden, da der Effekt der sozialen Erwünschtheit oft geringer sein wird. Bei stärker objektiven Fragen zur beruflichen oder familiären Situation der Befragten (‚Faktenfragen‘) sollte laut Studienergebnissen eine gute Vergleichbarkeit von CAWI mit persönlichen Interviews gegeben sein. Auch gibt es bei CAWI nicht unbedingt mehr Item-Non-Response („weiß nicht“ oder „keine Angabe“). CAWI kann jedoch zu einer höheren Rate an Survey-Non-Response führen, eine geringere Abdeckung der Zielpopulation (Internetzugang, digitale Affinität und Kompetenz) erreichen und damit einen größeren Stichprobenfehler bedingen (De Leeuw und Hox 2015). Zentral für die Einschätzung von Online-Befragungen ist die Art der Stichprobenziehung. CAWI auf Basis einer rein zufallsbasierten Auswahl von Befragten und einem adressbasierten *push-to-web* Design sollte einen deutlich kleineren Stichprobenfehler aufweisen als CAWI auf Basis von Online-Access Panels. Und hier gibt es in der Tat eine Reihe von methodischen Entwicklungen (Professionalisierung der *push-to-web* Designs, Befragungen über Mobiltelefone, Log-in für die Online-Befragung via QR Codes), die dazu führen, dass auch bei CAWI eine mit persönlichen Befragungen vergleichbare Survey Response Rate sowie eine hohe Repräsentativität der Stichprobe erreicht werden kann, wie beispielsweise bei der aktuellen Statistik-Austria Befragung „Wie geht’s uns heute“ (Mühlböck et al. 2022).

In diesem Zusammenhang ist zukünftige Forschung gefordert, ein stärkeres Augenmerk auf die Isolierung reiner *Messeffekte* (Effekte des Befragungsmodus per se), unter Kontrolle von *Selektionseffekten*, zu legen. Hier kann derzeit noch eine Forschungslücke ausgemacht werden. Selektionseffekte (unterschiedliche Zusammensetzung der Stichproben je nach Modus) können in der Tat größer ausfallen als die Messeffekte (Vannieuwenhuyze und Revilla 2013), die meist im Zentrum der Argumentation stehen. Zukünftige Forschung ist weiters gefordert, sich stärker mit *heterogenen* Moduseffekte zu beschäftigen, d. h. mit der Möglichkeit, dass sich der Modus der Befragung nicht auf alle Befragten gleich auswirkt. Heterogene Moduseffekte sind in der Literatur beschrieben, bleiben in der Praxis bis dato jedoch meist unbeachtet. Bei Erhebungen zur mentalen Gesundheit konnten beispielsweise unterschiedlich stark ausgeprägte Moduseffekte nach Bildung (Epstein Faith et al. 2001) und Alter (Wright et al. 1998) festgestellt werden. Auch bei politischen Einstellungen wurden unterschiedliche Moduseffekte nach Geschlecht, Alter und Bildung registriert (Sanders et al. 2007; Ansolabehere und Schaffner 2014). Obwohl es einige Evidenz dafür gibt, dass sich der Befragungsmodus nicht auf alle Bevölkerungsgruppen gleich auswirkt, blenden Theorien zum Antwortverhalten diese heterogenen Moduseffekte meist aus. Eine systematische Beschäftigung mit dem Thema ist noch ausständig (Pudney 2010; Heerwegh und Loosveldt 2011; Sánches Tome 2018, S. 153). Will man für Moduseffekte kontrollieren, um Trendanalyen auf Basis eines Vergleichs von persönlichen Interviews mit Online-Befragungen zu ermöglichen, ist es unabdingbar auf potenziell heterogene Moduseffekte zu achten. Die Kontrolle für homogene Moduseffekte kann – vor allem bei Gruppenvergleichen – womöglich zu stark verzerrten Resultaten führen (Jäckle et al. 2010; Backes und Cowan 2019).

### Supplementary Information





## Anhang

**Table 4:**

Themenbereich	Variable/Item	Frageformulierung und Ausprägungen oder Skala in Klammern
Finanzielle Lage	Subjektive Armutsgefährdung	Mit dem derzeitigen Haushaltseinkommen kann ich bzw. können wir … (1–bequem leben, 2–auskommen, 3–schwer auskommen, 4–nur sehr schwer auskommen, dichotomisiert in 1–bequem leben/auskommen)
	Ersparnisse/Schulden	Ich muss/wir müssen seit Beginn der Corona-Krise auf Ersparnisse zurückgreifen oder Schulden machen, um den normalen Lebensunterhalt zu bestreiten. (Skala von 1–trifft gar nicht zu bis 5–trifft voll und ganz zu)
	Zahlungsrückstände	Ich kann/wir können seit Beginn der Corona-Krise eine oder mehrere Forderungen/Rechnungen (z. B. Stromrechnung, Kreditrate, Miete, usw.) nicht termingerecht bezahlen. (Skala von 1–trifft gar nicht zu bis 5–trifft voll und ganz zu)
	Veränderung Verdienst	Wie hat sich Ihre berufliche Situation seit Beginn der Corona-Krise geändert? Wie viel ich verdiene/Mein Umsatz hat sich … (von 1–stark verringert bis 5–stark erhöht) (Zusammenfassung von zwei Fragen an Unselbstständige und Selbstständige)
	Subjektiver sozialer Status (0–10)	In unserer Gesellschaft gibt es Bevölkerungsgruppen, die eher „oben“ stehen, und solche, die eher „unten“ stehen. Wenn Sie an sich selbst denken: Wo würden Sie sich auf dieser Skala von 0–ganz unten bis 10–ganz oben einordnen?
Sorgen	Finanzielle Probleme	Wie viele Sorgen machen Sie sich, dass Sie aufgrund der Corona-Krise finanzielle Probleme bekommen? (Skala von 0–gar keine Sorgen bis 10–sehr große Sorgen)
	Gesundheitsversorgung	Wie viele Sorgen machen Sie sich, dass Sie aufgrund der Corona-Krise nicht die ärztliche Betreuung und Versorgung bekommen, die Sie brauchen? (Skala von 0–gar keine Sorgen bis 10–sehr große Sorgen)
	Einkommensverlust	Wie viele Sorgen machen Sie sich, dass Sie aufgrund der Corona-Krise Ihren Arbeitsplatz/Job verlieren? (Skala von 0–gar keine Sorgen bis 10–sehr große Sorgen)
	Jobverlust	Wie viele Sorgen machen Sie sich, dass Sie aufgrund der Corona-Krise Einkommenseinbußen erleiden? (Skala von 0–gar keine Sorgen bis 10–sehr große Sorgen)
Kinder	Home Schooling	Ich fühle mich durch die zusätzlichen Aufgaben für Eltern, die im Zusammenhang mit dem Lernen von zu Hause (Home Schooling) entstanden sind, überfordert. (Skala von 1–stimme voll und ganz zu bis 5–stimme überhaupt nicht zu)
	Sorge Lernfortschritt der Kinder	Ich mache mir Sorgen, dass sich das Lernen von zu Hause (Home Schooling) negativ auf den Lernfortschnitt meines Kindes/meiner Kinder auswirkt. (Skala von 1–stimme voll und ganz zu bis 5–stimme überhaupt nicht zu)
Gesundheit	Subjektive Gesundheit	Wie schätzen Sie Ihren allgemeinen Gesundheitszustand ein? (Skala von 1–sehr gut bis 5–sehr schlecht)
	CESD-Depressionsskala	Skala: Summenindex aus 8 Indikatoren zu psychischer Gesundheit – Liste zu Aussagen zur Häufigkeit von Gefühlen: Wie oft in letzte Woche (Skala von 1–nie oder fast nie bis 4–immer oder fast immer) 1–deprimiert oder niedergeschlagen gefühlt? 2–Gefühl gehabt, dass alles, was Sie getan haben, anstrengend war? 3–unruhig geschlafen? 4–waren Sie glücklich? 5–einsam gefühlt? 6–das Leben genossen? 7– traurig gefühlt? 8–Gefühl, isoliert oder ausgeschlossen zu sein?
Einstellungen	Einkommensumverteilung	Der Staat sollte Maßnahmen ergreifen, um Einkommensunterschiede zu reduzieren. (Skala von 1–stimme voll und ganz zu bis 5–stimme überhaupt nicht zu)
	Arbeitslosenunterstützung	Der Staat sollte für einen angemessenen Lebensstandard der Arbeitslosen sorgen. (Skala von 1–stimme voll und ganz zu bis 5–stimme überhaupt nicht zu)
	Armutsbekämpfung	Der Staat sollte viel mehr tun, damit die Leute nicht in Armut abgleiten. (Skala: von 1–stimme voll und ganz zu bis 5–stimme überhaupt nicht zu)
	Vermögensunterschiede	Der Staat sollte Maßnahmen ergreifen, um Vermögensunterschiede zu reduziere. (Skala: von 1–stimme voll und ganz zu bis 5–stimme überhaupt nicht zu)
	Unterschied Arm-Reich	Ich mache mir Sorgen, dass der Unterschied zwischen Arm und Reich aufgrund der Corona-Krise vergrößert wird. (Skala: von 1–stimme voll und ganz zu bis 5–stimme überhaupt nicht zu)
	Soziales Vertrauen	Würden Sie ganz generell sagen, dass man den meisten Menschen vertrauen kann oder, dass man im Umgang mit anderen Menschen nicht vorsichtig genug sein kann? Bitte wählen Sie Ihre Antwort von der Skala 0 bis 10, wobei 0 bedeutet, dass man nicht vorsichtig genug sein kann und 10 bedeutet, dass man den meisten Menschen vertrauen kann
Soziale Beziehungen	Zeit mit Familie	Und wie hat sich Ihre Paarbeziehung durch die Corona-Krise verändert? Die Zeit, die ich mit meiner Familie verbringe. (Skala von 1–stark verringert bis 5–stark erhöht)
	Zufriedenheit mit Beziehung	Und wie hat sich Ihre Paarbeziehung durch die Corona-Krise verändert? Meine Zufriedenheit mit der Beziehung. (Skala von 1–stark verringert bis 5–stark erhöht)
	Aufteilung Hausarbeit	Und wie hat sich Ihre Paarbeziehung durch die Corona-Krise verändert? Meine Zufriedenheit mit der Aufteilung der Hausarbeit. (Skala von 1–stark verringert bis 5–stark erhöht)
	Konflikt in Familie	Und wie hat sich Ihre Paarbeziehung durch die Corona-Krise verändert? Die Konflikte in meiner Familie/Beziehung. (Skala von 1–stark verringert bis 5–stark erhöht)
	Informelle Pflege vor Pandemie	Vor der Corona-Krise: Verbringen Sie Zeit damit, chronisch kranke, behinderte oder alte und pflegebedürftige Familienmitglieder, Freunde oder Nachbarn zu betreuen oder ihnen zu helfen? (Angabe in Stunden)
	Informelle Pflege in Pandemie	Derzeit: Verbringen Sie Zeit damit, chronisch kranke, behinderte oder alte und pflegebedürftige Familienmitglieder, Freunde oder Nachbarn zu betreuen oder ihnen zu helfen? (Angabe in Stunden)
Corona-Folgen	Kinderbetreuung	Die Kinderbetreuung stellt seit Beginn der Corona-Krise ein Problem für mich dar. (Skala von 1–stimme voll und ganz zu bis 5–stimme überhaupt nicht zu)
	Arbeitsstunden	Wie hat sich Ihre berufliche Situation seit Beginn der Corona-Krise geändert? Die Anzahl der Stunden, die ich pro Woche arbeite. (Skala von 1–stark verringert bis 5–stark erhöht)
	Zeit‑, Erfolgsdruck	Wie hat sich Ihre berufliche Situation seit Beginn der Corona-Krise geändert? Der Zeit- oder Erfolgsdruck in der Arbeit. (Skala von 1–stark verringert bis 5–stark erhöht)
	Home-Office	Wie hat sich Ihre berufliche Situation seit Beginn der Corona-Krise geändert? Wie viel ich von zu Hause aus arbeite (Skala von 1–stark verringert bis 5–stark erhöht)
	Anerkennung	Wie hat sich Ihre berufliche Situation seit Beginn der Corona-Krise geändert? Die gesellschaftliche Anerkennung für meine Tätigkeit (Skala von 1–stark verringert bis 5–stark erhöht)
	Autonomie	Wie hat sich Ihre berufliche Situation seit Beginn der Corona-Krise geändert? Wie sehr ich Entscheidungen, die wichtig für meine Arbeit sind, beeinflussen kann. (Skala von 1–stark verringert bis 5–stark erhöht)
	Sicherheit Job	Wie hat sich Ihre berufliche Situation seit Beginn der Corona-Krise geändert? Die Sicherheit meines Arbeitsplatzes (Skala von 1–stark verringert bis 5–stark erhöht)
	Planung Arbeit	Wie hat sich Ihre berufliche Situation seit Beginn der Corona-Krise geändert? Wie sehr ich meine Arbeit selbst planen/einteilen kann (Skala von 1–stark verringert bis 5–stark erhöht)
	Überwachung im Job	Wie hat sich Ihre berufliche Situation seit Beginn der Corona-Krise geändert? Wie stark meine Arbeit überwacht wird (Skala von 1–stark verringert bis 5–stark erhöht)
	Persönlicher Kontakt im Job	Wie hat sich Ihre berufliche Situation seit Beginn der Corona-Krise geändert? Der persönliche Kontakt zu Menschen (Kollegen(innen), Vorgesetzte, Kunden(innen), Klient(innen), Patienten(innen), Lehrlinge, Schüler/Studierende, usw.) (Skala von 1–stark verringert bis 5–stark erhöht)
	Vereinbarkeit	Würden Sie sagen, dass es seit Beginn der Corona-Krise einfacher oder schwieriger geworden ist, Beruf und Familie miteinander zu vereinbaren? (Skala 1–einfacher, 2–gleich, 3–schwieriger, dichotomisiert in 1 =schwieriger) (ein ordinales logistisches Modell mit drei Kategorien führt zu vergleichbaren Ergebnissen)
	Infektionsrisiko am Arbeitsplatz	Wie hoch schätzen Sie das Risiko ein, dass Sie sich im Rahmen Ihrer beruflichen Tätigkeit mit dem Corona-Virus anstecken? (Skala 1–sehr hoch bis 4–sehr niedrig, dichotomisiert in 1 =hoch) (ordinales logistisches Modell mit vergleichbaren Ergebnissen)
Arbeitsbedingungen	Betriebsrat vorhanden	Gibt es in Ihrem Betrieb bzw. in der Organisation, für die Sie arbeiten, einen Betriebsrat? (0–nein, 1–ja)
	Home-Office	Wie oft arbeiten Sie derzeit von zu Hause aus? (von 1–täglich bis 5–nie)
	Arbeitsstunden	Wie viele Stunden arbeiten Sie derzeit durchschnittlich pro Woche? (Angabe in Stunden)
	Wissen Vorgesetzte	Wie einfach oder schwierig ist es für Ihre direkten Vorgesetzten zu wissen, wie groß Ihr Einsatz bei der Arbeit ist? (Skala von 0–äußerst einfach bis 10–äußerst schwierig)
	Autonomie im Job	Skala: Summenindex aus vier Indikatoren zu Arbeitsautonomie (von 1–keine Selbstbestimmung bis 5–starke Selbstbestimmung)
	Tätigkeit Probleme lösen	Wie häufig schließt Ihre derzeitige berufliche Tätigkeit Folgendes ein? das selbständige Lösen unvorhergesehener Probleme (Skala von 1–immer bis 6–nie)
	Tätigkeit eintönig	Wie häufig schließt Ihre derzeitige berufliche Tätigkeit Folgendes ein? eintönige Aufgaben (Skala von 1–immer bis 6–nie)
	Tätigkeit Dinge lernen	Wie häufig schließt Ihre derzeitige berufliche Tätigkeit Folgendes ein? das Lernen neuer Dinge (Skala von 1–immer bis 6–nie)
	Tätigkeit eigene Ideen	Wie häufig schließt Ihre derzeitige berufliche Tätigkeit Folgendes ein? das Umsetzen meiner eigenen Ideen (Skala von 1–immer bis 6–nie)

## Footnotes

1 In Österreich wird bei CAWI auf Basis eines Online-Access Panels meist das Marktforschungsinstitut *Marketagent* beauftragt. Dieses Online-Access Panel umfasst rund 130 Tausend Personen und erfüllt die Anforderung an Strukturgleichheit zur österreichischen Wohnbevölkerung ab einem Alter von 14 Jahren. Die Rekrutierung der Panelteilnehmenden erfolgt über verschiedene Kanäle (z. B. Fernsehwerbung, Werbeplakate, Inserate in Zeitschriften). Die Teilnehmenden erhalten Incentives für die Teilnahme an Befragungen (z. B. Gutscheinen oder auch Bezahlung für konkrete Befragungen).

2 Items werden als quasi-metrisch interpretiert, wenn diese mindestens fünf Ausprägungen aufweisen. Items mit fünf oder sechs Ausprägungen weisen eine vollständige symmetrische Beschriftung der Antwortoptionen auf; jene mit mehr Ausprägungen beschriftete Randkategorien mit numerischen Abstufungen. Es wird angenommen, dass die Variablen das Kriterium gleicher Abstände erfüllen (Baur 2008; Urban und Mayerl 2011; Völkl und Korb 2018). Aus den acht Items zur mentalen Gesundheit gemäß CES‑D Depressionsskala wurde ein Summenindex erstellt (Van de Velde et al. 2009).

3 Zusätzlich wurden bei den ordinalen Variablen die Schnittpunkte zur Dichotomisierung variiert. Einzig der Moduseffekt bei der Frage zu subjektiver Armutsgefährdung (Tab. 2 und 4) ist durch die Verwendung anderer Schnittpunkte für die Dichotomisierung nicht mehr signifikant (aufgrund der asymmetrischen Verteilung der binären Variablen). Allerdings weist eine ordinale logistische Regression einen signifikanten Moduseffekt aus, weshalb wir die Ergebnisse in Tab. 1 auch bei dieser Frage als belastbar ansehen und so der Trend im Fragenblock Finanzielle Lage bestätigt wird (siehe Abschn. 4.3).

4 Es kann nur für Unterschiede in der Stichprobenzusammensetzung von CATI und CAWI kontrolliert werden, insofern sich die Unterschiede auf in der Befragung gemessene Eigenschaften beziehen. Es kann nicht ausgeschlossen werden, dass sich die beiden Stichproben anhand weiterer Eigenschaften voneinander unterscheiden, die das Antwortverhalten beeinflussen (siehe Abschn. 2.1).

5 Arbeitszeit, Arbeitsdruck, betriebliche Mitbestimmung, Arbeitsautonomie, Arbeitsüberwachung, persönliche Kontakte zu Menschen, gefühlte gesellschaftliche Anerkennung für die berufliche Tätigkeit, sowie die Einschätzung des Risikos sich bei der Arbeit mit dem Coronavirus anzustecken.

6 Auf Basis eines Regressionsmodells analog zu jenem im Online-Supplement für dieses Item, jedoch anhand eines nicht standardisierten Regressionskoeffizienten.
