# Supplementary material for: Do online interviews lead to different results compared to personal interviews? Estimates of mode effects using a mixed mode survey
Source: Österreich Z Soziol. 2023 Jun 5:1–22. [Article in German] Online ahead of print. doi: 10.1007/s11614-023-00532-4 (PMC10243093; doi:10.1007/s11614-023-00532-4)
Supplement: Supplementary file 1 [file 11614_2023_532_MOESM1_ESM.pdf]

Liedl/Steiber (2023): Führen Online-Befragungen zu anderen Ergebnissen als persönliche Interviews?

Online-Supplement: Tabellenband

Themenbereich	Variable/Item (i)	Moduseffekte (ii) (iii)	Effektstärke: Beta (iv)	Effektstärke: Eta Quadrat (v)	Heterogene Effekte je nach Merkmal (vi)
**Finanzielle Lage**	Subjektive Armutsgefährdung	CAWI-schwer zurechtkommen	-0,12***	0,009 +	
(siehe Reiter M-Finanzen	Finanzielle Lage: Ersparnisse/Schulden	CAWI-stärkere Zustimmung	0,10***	0,014 ++	
für Modelle mit Kovariaten)	Zahlungsrückstände	CAWI-stärkere Zustimmung	0,07***	0,008 +	
	Veränderung Verdienst wegen Pandemie		-0,01		
	Subjektiver sozialer Status (0-10)	CATI-höher (CATI-mehr non-response)	-0,04**	0,007 +	
**Sorgen**	Finanzielle Probleme	CAWI-mehr Sorgen	0,10***	0,013 ++	
(siehe Reiter M-Sorgen	Gesundheitsversorgung	CAWI-mehr Sorgen	0,06**	0,005 +	Alter
für Modelle mit Kovariaten)	Einkommensverlust	CAWI-mehr Sorgen	0,08***	0,011 ++	
	Jobverlust	CAWI-mehr Sorgen	0,10***	0,013 ++	
**Kinder**	Überforderung durch Home Schooling	CAWI-mehr Überforderung	-0,12***	0,015 ++	
(siehe Reiter M-Kinder)	Sorge Lernfortschritt der Kinder	CAWI-mehr Sorgen	-0,13***	0,016 ++	
**Gesundheit**	subjektive Gesundheit	CATI-gesünder (CAWI-mehr non-response)	0,04**	0,005 +	Alter
(siehe Reiter M-Gesundheit)	CESD-Depressionsskala	CATI-gesünder	0,05***	0,01 ++	
**Einstellungen**	Einkommensumverteilung		-0,02		
(siehe Reiter M-Einstellungen	Arbeitslosenunterstützung	CATI-stärkere Zustimmung	0,06**	0,005 +	Bildung, Alter
für Modelle mit Kovariaten)	Armutsbekämpfung		0,02		Bildung
	Vermögensunterschiede	(CAWI-mehr non-response)	-0,02		Bildung
	Unterschied Arm-Reich	(CAWI-mehr non-response)	-0,00		Bildung
	Soziales Vertrauen	CATI-mehr Vertrauen	-0,14***	0,041 ++	Alter
**Soziale Beziehungen**	Zeit mit Familie		0,00		
(siehe Reiter M-SozBezi	Zufriedenheit mit Beziehung		-0,01		
für Modelle mit Kovariaten)	Zufriedenheit mit Aufteilung Hausarbeit		-0,02		Alter
	Konflikt in Familie		0,01		
	Informelle Pflege vor der Pandemie		-0,01		
	Informelle Pflege in der Pandemie		-0,00		
**Corona-Folgen**	Kinderbetreuung		-0,04		
(siehe Reiter M-Corona-Folgen	Arbeitsstunden		-0,03		
für Modelle mit Kovariaten)	Zeit-, Erfolgsdruck		-0,03		
	Home Office		0,01		
	Anerkennung		-0,03		
	Autonomie		-0,02		
	Sicherheit Job	CATI-erhöht	-0,05**	0,005 +	
	Planung Arbeit		-0,00		
	Überwachung im Job		0,00		
	Persönlicher Kontakt im Job		0,00		
	Vereinbarkeit		-0,02		
	Infektionsrisiko am Arbeitsplatz		0,02		
**Arbeitsbedingungen**	Betriebsrat vorhanden	(CAWI-mehr non-response)	0,01		
(siehe Reiter M-Arbeit	Home Office		-0,01		
für Modelle mit Kovariaten)	Arbeitsstunden	(CAWI-mehr non-response)	-0,01		Bildung
	Wissen Vorgesestzte	(CATI-mehr non-response)	0,04		
	Autonomie im Job		0,00		
	Tätigkeit Probleme lösen		-0,01		
	Tätigkeit eintönig		-0,04		Bildung
	Tätigkeit Dinge lernen		0,02		Gender
	Tätigkeit eigene Ideen		0,01		Gender

Methoden der Auswertung: Bei (quasi)metrischen Variablen lineare Regression (OLS), bei ordinalen Variablen wurden diese dichotomisiert und mittels linearer Wahrscheinlichkeitsmodelle modelliert.
(i) Für genaues Wording der Items im Fragebogen, siehe folgende Seiten mit Regressionstabellen
(ii) In der Spalte werden die Moduseffekte berichtet, wenn ein statistisch signifikanter Effekt im Regressionsmodell auftritt.
(iii) Bewertung der Non-Response: Überlappen die Vertrauensintervalle der Anteile der Missings nicht, wird der Modus mit mehr Missings in Klammern ausgewiesen.
*(iv) Effektstärke laut Regressionsmodell in Form von standardisierten Koeffizienten (und deren Signifikanzniveau: * p<0,05 ** p<0,01 *** p<0,001)*
(v) Effektstärker laut Regressionsmodell in Form von Eta Quadrat (+ sehr kleiner Effekt ++ kleiner Effekt), Richtung des Effekts von Spalte 'Moduseffekte' abzulesen.
(vi) Heterogene Effekte wurden als Interaktion des Modus mit dem genannten Merkmal identifiziert.
Es werden nur heterogene Effekte ausgewiesen, die einen signifikanten Interaktionseffekt mit dem Modus zeigen und wenn auch in getrennten Modellen zumindest
für eine der beiden Ausprägungen der dichotomisierten Variablen (Bildung mit Matura=1, Altersgruppe mit 1=55 und älter und Geschlecht mit 1=Frau) ein signifikanter Haupteffekt vorliegt.

**Themenbereich: Finanzielle Lage**	**Subjektive Armuts-gefährdung**	**Finanzielle Lage: Ersparnisse/ Schulden**	**Zahlungs-rückstände**	**Veränderung Verdienst wegen Pandemie**	**Subjektiver sozialer Status**
	(1)++	(2)+	(3)+	(4)+	(5)+
Gender (Ref.: Männer)					
Frauen	0.01	0.02	0.02	0.01	-0.01
Alter (Ref.: 20-29)					
30-39	-0.05	0.02	-0.02	-0.02	0.00
40-49	0.02	-0.03	-0.08***	-0.02	0.01
50-59	0.00	-0.04	-0.08***	-0.08***	0.02
60-64	0.05	-0.15***	-0.16***	-0.09*	0.07**
Bildung (Ref.: Pflichtschule)					
Lehre	0.08	-0.01	-0.01	0.02	0.02
BMS	0.13*	-0.05	-0.01	0.01	0.04
AHS-Matura	0.21**	-0.02	-0.03	0.02	0.08**
BHS-Matura	0.22***	-0.05	-0.07*	0.01	0.05*
Diplom, Univ.-Lehrgang	0.25***	-0.05	-0.08*	0.01	0.09**
Hochschule	0.28***	-0.08	-0.08*	-0.02	0.11***
Bundesland (Ref.: Vorarlberg)					
Tirol	0.09	0.01	-0.04	-0.00	0.02
Salzburg	-0.00	0.06	0.02	-0.04	-0.03
Oberösterreich	0.04	-0.01	-0.04	0.02	-0.01
Kärnten	0.08	0.04	-0.00	0.05	-0.02
Steiermark	0.02	0.02	-0.02	-0.00	-0.04
Burgenland	-0.04	0.04	0.02	0.10*	-0.02
Niederösterreich	0.03	-0.00	-0.04	0.01	-0.02
Wien	0.01	0.05	0.04	-0.01	-0.01
Wohnort (Ref.: Land)					
Größeres Dorf/ Kleinstadt	-0.01	0.00	-0.02	0.04*	0.01
Mittelstadt	-0.02	0.02	-0.00	0.02	0.01
Großstadt/ Vorstadt	0.03	-0.03	-0.05*	0.04	-0.01
Staatsbürgerschaft					
AUT	0.16**	-0.09*	-0.12***	-0.02	0.01
Tätigkeit (Ref.: Vollzeit)					
Teilzeit	-0.06	-0.01	-0.02	0.00	0.00
Kurzarbeit	-0.25***	0.20***	0.08**	-0.18***	-0.06**
Ausbildung	-0.03	0.02	0.06		-0.07
Arbeitslos	-0.28***	0.22***	0.15***		-0.12***
Karenz, Hausarbeit	-0.23***	0.10**	-0.01	-0.03	-0.06**
Pension, Arbeitsunfähig	-0.11*	0.10**	0.08**		-0.10***
Mode (Ref.: CATI)					
CAWI	-0.12***	0.10***	0.07***	-0.01	-0.04**
_cons	0.14	0.24***	0.29***	0.49***	0.54***
N	1410	1407	1410	1069	1348
R-sq	0.124	0.125	0.110	0.116	0.128

Quelle: ANONYM-Survey, Daten erhoben im Jänner 2021. Sample: in Österreich lebende Personen im Alter von 20-64.

+ Lineare Regressionsmodelle; die abhängigen Variablen wurden auf den Wertebereich 0 bis 1 standardisiert.

++ Lineares Wahrscheinlichkeitsmodell; die abhängige Variable wurde dichotomisiert.

**** p<0.001; ** p<0.01; * p<0.05*

(1) Mit dem derzeitigen Haushaltseinkommen kann ich bzw. können wir… (Skala von 1-bequem leben, 2-auskommen, 3-schwer auskommen, 4-nur sehr schwer auskommen, dichotomisiert in 1-bequem leben/auskommen)

(2) Ich muss/wir müssen seit Beginn der Corona-Krise auf Ersparnisse zurückgreifen oder Schulden machen, um den normalen Lebensunterhalt zu bestreiten. (Skala von 1-trifft gar nicht zu bis 5-trifft voll und ganz zu)

(3) Ich kann/wir können seit Beginn der Corona-Krise eine oder mehrere Forderungen/ Rechnungen (z.B. Stromrechnung, Kreditrate, Miete, usw.) nicht termingerecht bezahlen. (Skala von 1-trifft gar nicht zu bis 5-trifft voll und ganz zu)

(4) Wie hat sich Ihre berufliche Situation seit Beginn der Corona-Krise geändert? Wie viel ich verdiene/Mein Umsatz hat sich… (von 1-stark verringert bis 5-stark erhöht) (Zusammenfassung von zwei Fragen an Unselbstständige und Selbstständige)

(5) In unserer Gesellschaft gibt es Bevölkerungsgruppen, die eher „oben" stehen, und solche, die eher „unten" stehen. Wenn Sie an sich selbst denken: Wo würden Sie sich auf dieser Skala von 0-ganz unten bis 10-ganz oben einordnen?

Themenbereich: Sorgen	Finanzielle Pobleme	Gesundheits-versorgung	Einkommens-verlust	Jobverlust
	(1)	(2)	(3)	(4)
Gender (Ref.: Männer)				
Frauen	0.05**	0.05**	-0.01	-0.01
Alter (Ref.: 20-29)				
30-39	0.03	0.05*	0.01	0.01
40-49	0.03	0.07**	0.03	0.01
50-59	0.02	0.06*	0.04	0.03
60-64	-0.13***	-0.03	-0.03	-0.06
Bildung (Ref.: Pflichtschule)				
Lehre	-0.00	-0.05	-0.03	0.05
BMS	-0.03	-0.11**	-0.03	-0.00
AHS-Matura	-0.06	-0.07	0.01	0.02
BHS-Matura	-0.08*	-0.09*	-0.06	-0.05
Diplom, Univ.-Lehrgang	-0.12**	-0.13**	-0.06	-0.04
Hochschule	-0.06	-0.13***	-0.02	0.01
Bundesland (Ref.: Vorarlberg)				
Tirol	-0.00	-0.09*	-0.04	0.00
Salzburg	0.11*	0.03	0.11*	0.10
Oberösterreich	0.02	-0.02	0.03	0.07
Kärnten	0.04	-0.06	-0.03	0.01
Steiermark	0.03	0.00	0.02	0.06
Burgenland	0.06	0.04	-0.01	0.11
Niederösterreich	0.02	-0.03	0.01	0.05
Wien	-0.02	-0.02	0.04	0.03
Wohnort (Ref.: Land)				
Größeres Dorf/ Kleinstadt	0.03	0.01	0.03	0.02
Mittelstadt	0.00	-0.01	0.01	-0.01
Großstadt/ Vorstadt	0.05	0.00	-0.02	0.02
Staatsbürgerschaft				
AUT	-0.06*	-0.06*	-0.10**	-0.09*
Tätigkeit (Ref.: Vollzeit)				
Teilzeit	-0.02	0.00	-0.01	-0.02
Kurzarbeit	0.17***	0.06**	0.15***	0.27***
Ausbildung	-0.06	-0.05		
Arbeitslos	0.22***	0.10**		
Karenz, Hausarbeit	0.07*	0.06	0.14**	0.07
Pension, Arbeitsunfähig	0.09**	0.15***		
Mode (Ref.: CATI)				
CAWI	0.10***	0.06**	0.08***	0.10***
_cons	0.28***	0.37***	0.22***	0.26***
N	1960	1911	1495	1500
R-sq	0.127	0.072	0.084	0.136

Quelle: ANONYM-Survey, Daten erhoben im Juni 2020. Sample: in Österreich lebende Personen im Alter von 20-64.
Lineare Regressionsmodelle; die abhängigen Variablen wurden auf den Wertebereich 0 bis 1 standardisiert.
**** p<0.001; ** p<0.01; * p<0.05*

(1) Wie viele Sorgen machen Sie sich, dass Sie aufgrund der Corona-Krise finanzielle Probleme bekommen? (Skala von 0-gar keine Sorgen bis 10-sehr große Sorgen)
(2) Wie viele Sorgen machen Sie sich, dass Sie aufgrund der Corona-Krise nicht die ärztliche Betreuung und Versorgung bekommen, die Sie brauchen? (Skala 0-10)
(3) Wie viele Sorgen machen Sie sich, dass Sie aufgrund der Corona-Krise Ihren Arbeitsplatz/Job verlieren? (Skala von 0-gar keine Sorgen bis 10-sehr große Sorgen)
(4) Wie viele Sorgen machen Sie sich, dass Sie aufgrund der Corona-Krise Einkommenseinbußen erleiden? (Skala von 0-gar keine Sorgen bis 10-sehr große Sorgen)

Themenbereich: Kinder	Überforderung durch Home Schooling	Sorge Lernfortschritt der Kinder
	(1)	(2)
Gender (Ref.: Männer)		
Frauen	-0.04	-0.04
Alter (Ref.: 20-29)		
30-39	-0.13**	-0.16**
40-49	-0.04	-0.17**
50-59	0.07	-0.08
60-64	0.28	0.02
Bildung (Ref.: Pflichtschule)		
Lehre	-0.05	-0.08
BMS	-0.01	-0.02
AHS-Matura	-0.06	0.01
BHS-Matura	0.01	-0.01
Diplom, Univ.-Lehrgang	-0.04	0.01
Hochschule	0.03	0.03
Bundesland (Ref.: Vorarlberg)		
Tirol	-0.02	-0.07
Salzburg	0.00	-0.05
Oberösterreich	0.08	0.02
Kärnten	0.11	0.09
Steiermark	0.04	-0.05
Burgenland	-0.05	0.05
Niederösterreich	0.05	0.01
Wien	-0.02	-0.06
Wohnort (Ref.: Land)		
Größeres Dorf/ Kleinstadt	-0.06	-0.06
Mittelstadt	-0.03	-0.04
Großstadt/ Vorstadt	-0.05	-0.03
Staatsbürgerschaft		
AUT	0.03	0.04
Tätigkeit (Ref.: Vollzeit)		
Teilzeit	-0.05	-0.02
Kurzarbeit	-0.02	-0.02
Ausbildung	-0.06	-0.10
Arbeitslos	-0.06	-0.13*
Karenz, Hausarbeit	0.07	-0.02
Pension, Arbeitsunfähig	-0.14	-0.08
Mode (Ref.: CATI)		
CAWI	-0.12***	-0.13***
_cons	0.71***	0.81***
N	701	702
R-sq	0.115	0.092

Quelle: ANONYM-Survey, Daten erhoben im Juni 2020. Sample: in Österreich lebende Personen im Alter von 20-64.
Lineare Regressionsmodelle; die abhängigen Variablen wurden auf den Wertebereich 0 bis 1 standardisiert.
**** p<0.001; ** p<0.01; * p<0.05*

(1) Ich fühle mich durch die zusätzlichen Aufgaben für Eltern, die im Zusammenhang mit dem Lernen von zu Hause (Home Schooling) entstanden sind, überfordert.
(2) Ich mache mir Sorgen, dass sich das Lernen von zu Hause (Home Schooling) negativ auf den Lernfortschritt meines Kindes/meiner Kinder auswirkt.
Skala jeweils von von 1-stimme voll und ganz zu bis 5-stimme überhaupt nicht zu

Themenbereich: Gesundheit	subjektive Gesundheit	CESD-Depressions-skala
	(1)	(2)
Gender (Ref.: Männer)		
Frauen	-0.03**	0.03**
Alter (Ref.: 20-29)		
30-39	0.00	-0.01
40-49	0.03*	-0.03
50-59	0.07***	-0.02
60-64	-0.03	-0.13***
Bildung (Ref.: Pflichtschule)		
Lehre	-0.04*	-0.07***
BMS	-0.06**	-0.10***
AHS-Matura	-0.05*	-0.06*
BHS-Matura	-0.07**	-0.10***
Diplom, Univ.-Lehrgang	-0.07*	-0.09***
Hochschule	-0.08***	-0.08***
Bundesland (Ref.: Vorarlberg)		
Tirol	-0.03	-0.06*
Salzburg	-0.00	-0.04
Oberösterreich	-0.01	-0.04
Kärnten	-0.02	-0.05
Steiermark	-0.01	-0.04
Burgenland	0.05	-0.03
Niederösterreich	0.02	-0.04
Wien	0.01	0.01
Wohnort (Ref.: Land)		
Größeres Dorf/ Kleinstadt	0.02	0.02
Mittelstadt	0.02	0.03*
Großstadt/ Vorstadt	-0.00	0.00
Staatsbürgerschaft		
AUT	-0.00	-0.04*
Tätigkeit (Ref.: Vollzeit)		
Teilzeit	0.03	0.00
Kurzarbeit	0.02	0.03*
Ausbildung	0.05	0.03
Arbeitslos	0.14***	0.10***
Karenz, Hausarbeit	0.06**	0.03
Pension, Arbeitsunfähig	0.21***	0.11***
Mode (Ref.: CATI)		
CAWI	0.04**	0.05***
_cons	0.23***	0.35***
N	1970	1921
R-sq	0.135	0.104

Quelle: ANONYM-Survey, Daten erhoben im Juni 2020. Sample: in Österreich lebende Personen im Alter von 20-64.
Lineare Regressionsmodelle; die abhängigen Variablen wurden auf den Wertebereich 0 bis 1 standardisiert.
**** p<0.001; ** p<0.01; * p<0.05*

(1) Wie schätzen Sie Ihren allgemeinen Gesundheitszustand ein? (Skala von 1-sehr gut bis 5-sehr schlecht)
(2) Skala: Summenindex aus 8 Indikatoren zu psychischer Gesundheit (CESD-Skala) (Skala von 1-nie oder fast nie bis 5-fast immer)

Themenbereich: Einstellungen	Einkommens-umverteilung	Arbeitslosen-unterstützung	Armuts-bekämpfung	Vermögens-unterschiede	Unterschied Arm-Reich	Soziales Vertrauen
	(1)	(2)	(3)	(4)	(5)	(6)
Gender (Ref.: Männer)						
Frauen	-0.03*	0.04**	-0.00	-0.02	-0.02	-0.01
Alter (Ref.: 20-29)						
30-39	-0.00	0.06**	-0.00	0.02	-0.01	-0.00
40-49	0.03	0.07***	-0.01	0.04	-0.03	0.00
50-59	0.01	0.02	-0.02	0.04	-0.05*	0.01
60-64	0.04	0.03	0.02	0.03	-0.07*	0.02
Bildung (Ref.: Pflichtschule)						
Lehre	-0.02	0.04	0.01	0.02	-0.05	-0.00
BMS	0.01	0.02	0.02	0.04	-0.00	0.03
AHS-Matura	0.04	-0.01	0.04	0.10**	0.01	0.07
BHS-Matura	0.04	0.01	0.03	0.08*	0.00	0.06
Diplom, Univ.-Lehrgang	0.04	-0.03	0.02	0.06	-0.03	0.12**
Hochschule	0.06*	-0.01	0.02	0.08*	-0.00	0.10**
Bundesland (Ref.: Vorarlberg)						
Tirol	0.04	0.00	-0.01	0.02	0.01	0.02
Salzburg	0.08*	0.02	-0.00	0.07	0.02	-0.00
Oberösterreich	0.05	0.04	0.01	0.03	0.01	0.00
Kärnten	0.08*	0.05	-0.01	0.03	0.02	0.01
Steiermark	0.04	0.05	-0.01	0.05	0.02	-0.01
Burgenland	0.04	0.08	0.03	0.05	-0.01	-0.04
Niederösterreich	0.05	0.01	-0.01	0.03	0.00	-0.05
Wien	0.06	0.00	0.01	0.05	0.03	-0.01
Wohnort (Ref.: Land)						
Größeres Dorf/ Kleinstadt	-0.01	-0.01	-0.01	-0.01	-0.01	-0.00
Mittelstadt	-0.02	-0.03	-0.03	-0.01	-0.04*	-0.00
Großstadt/ Vorstadt	-0.04	-0.01	-0.03	-0.04	-0.03	-0.03
Staatsbürgerschaft						
AUT	-0.00	0.11***	0.01	0.03	0.02	-0.01
Tätigkeit (Ref.: Vollzeit)	0.00	0.00	0.00	0.00	0.00	0.00
Teilzeit	-0.03	-0.02	-0.03	0.00	-0.03	0.00
Kurzarbeit	-0.01	-0.03	0.00	-0.00	-0.01	0.02
Ausbildung	-0.01	-0.11*	-0.09*	-0.00	0.04	-0.03
Arbeitslos	-0.05	-0.20***	-0.12***	-0.04	-0.06*	-0.04
Karenz, Hausarbeit	0.01	-0.03	-0.04	0.01	-0.04	-0.01
Pension, Arbeitsunfähig	-0.05*	-0.06*	-0.06**	-0.03	-0.02	0.01
Mode (Ref.: CATI)						
CAWI	-0.02	0.06**	0.02	-0.02	-0.00	-0.14***
_cons	0.23***	0.21***	0.24***	0.19***	0.29***	0.55***
N	1931	1910	1941	1902	1933	1390
R-sq	0.032	0.076	0.028	0.023	0.027	0.093

Quelle: ANONYM-Survey, Modelle (1)-(5): Daten erhoben im Juni 2020, Modell (6): Jänner 2021. Sample: in Österreich lebende Personen im Alter von 20-64.
Lineare Regressionsmodelle; die abhängigen Variablen wurden auf den Wertebereich 0 bis 1 standardisiert.
**** p<0.001; ** p<0.01; * p<0.05*

(1) Der Staat sollte Maßnahmen ergreifen, um Einkommensunterschiede zu reduzieren. (Skala: von 1-stimme voll und ganz zu bis 5-stimme überhaupt nicht zu)
(2) Der Staat sollte für einen angemessenen Lebensstandard der Arbeitslosen sorgen. (Skala: von 1-stimme voll und ganz zu bis 5-stimme überhaupt nicht zu)
(3) Der Staat sollte viel mehr tun, damit die Leute nicht in Armut abgleiten. (Skala: von 1-stimme voll und ganz zu bis 5-stimme überhaupt nicht zu)
(4) Der Staat sollte Maßnahmen ergreifen, um Vermögensunterschiede zu reduziere. (Skala: von 1-stimme voll und ganz zu bis 5-stimme überhaupt nicht zu)
(5) Ich mache mir Sorgen, dass der Unterschied zwischen Arm und Reich aufgrund der Corona-Krise vergrößert wird. (Skala: von 1-stimme voll und ganz zu bis 5-stimme überhaupt nicht zu)
(6) Würden Sie ganz generell sagen, dass man den meisten Menschen vertrauen kann oder, dass man im Umgang mit anderen Menschen nicht vorsichtig genug sein kann?
Bitte wählen Sie Ihre Antwort von der Skala 0 bis 10, wobei 0 bedeutet, dass man nicht vorsichtig genug sein kann und 10 bedeutet, dass man den meisten Menschen vertrauen kann.

Themenbereich: Soziale Beziehungen	Zeit mit Familie	Zufriedenheit mit Beziehung	Zufriedenheit mit Aufteilung Hausarbeit	Konflikt in Familie	Informelle Pflege vor der Pandemie	Informelle Pflege in der Pandemie
	(1)	(2)	(3)	(4)	(5)	(6)
Gender (Ref.: Männer)						
Frauen	-0.04*	0.01	-0.03*	-0.01	0.01	-0.00
Alter (Ref.: 20-29)						
30-39	0.05	-0.01	-0.02	0.00	-0.02	-0.01
40-49	0.08**	-0.01	-0.02	-0.01	-0.03	-0.02
50-59	0.04	-0.01	-0.02	-0.02	-0.06*	-0.04
60-64	-0.01	-0.01	-0.04	-0.03	-0.05	-0.05
Bildung (Ref.: Pflichtschule)						
Lehre	0.02	-0.02	-0.01	0.00	-0.05	-0.03
BMS	0.06	-0.02	-0.02	-0.01	-0.03	-0.03
AHS-Matura	0.01	-0.05	-0.04	0.04	-0.03	-0.06
BHS-Matura	0.10**	-0.00	-0.02	-0.00	-0.07	-0.06
Diplom, Univ.-Lehrgang	0.04	0.01	-0.04	0.01	-0.03	-0.00
Hochschule	0.09*	-0.03	-0.05*	0.02	-0.01	-0.00
Bundesland (Ref.: Vorarlberg)						
Tirol	0.01	0.03	0.02	-0.00	0.01	-0.03
Salzburg	0.03	0.01	-0.02	-0.01	0.08	0.04
Oberösterreich	0.03	0.02	0.01	-0.01	0.06	0.04
Kärnten	0.02	0.05	0.02	-0.00	0.04	0.05
Steiermark	0.05	0.02	-0.01	-0.01	0.11*	0.07
Burgenland	-0.03	0.01	-0.01	0.01	0.03	-0.03
Niederösterreich	-0.00	0.02	0.00	-0.00	0.04	-0.00
Wien	-0.05	0.03	0.01	-0.03	0.08	0.03
Wohnort (Ref.: Land)						
Größeres Dorf/ Kleinstadt	0.04*	0.01	0.01	-0.00	-0.00	-0.02
Mittelstadt	-0.03	-0.00	0.01	-0.02	0.01	-0.03
Großstadt/ Vorstadt	0.06*	-0.02	0.01	0.03	-0.04	-0.03
Staatsbürgerschaft						
AUT	0.04	0.01	-0.01	0.04	-0.03	-0.05
Tätigkeit (Ref.: Vollzeit)						
Teilzeit	0.07**	0.01	0.01	0.02	-0.03	-0.02
Kurzarbeit	0.09***	0.02	-0.00	0.03	0.02	-0.02
Ausbildung	-0.04	0.13*	0.06	0.03	-0.06	-0.05
Arbeitslos	0.08**	0.02	0.03	0.02	-0.03	-0.01
Karenz, Hausarbeit	0.09**	-0.00	0.02	0.08***	0.09*	0.11**
Pension, Arbeitsunfähig	-0.06	-0.01	0.03	0.04	0.01	0.01
Mode (Ref.: CATI)						
CAWI	0.00	-0.01	-0.02	0.01	-0.01	-0.00
_cons	0.49***	0.54***	0.57***	0.46***	0.14	0.17*
N	1350	1347	1347	1319	262	276
R-sq	0.073	0.015	0.020	0.028	0.157	0.164

Quelle: ANONYM-Survey, Daten erhoben im Juni 2020. Sample: in Österreich lebende Personen im Alter von 20-64.

Lineare Regressionsmodelle; die abhängigen Variablen wurden auf den Wertebereich 0 bis 1 standardisiert.

**** p<0.001; ** p<0.01; * p<0.05*

(1) Und wie hat sich Ihre Paarbeziehung durch die Corona-Krise verändert? Die Zeit, die ich mit meiner Familie verbringe. (Skala von 1-stark verrringert bis 5-stark erhöht)
(2) Und wie hat sich Ihre Paarbeziehung durch die Corona-Krise verändert? Meine Zufriedenheit mit der Beziehung. (Skala von 1-stark verrringert bis 5-stark erhöht)
(3) Und wie hat sich Ihre Paarbeziehung durch die Corona-Krise verändert? Meine Zufriedenheit mit der Aufteilung der Hausarbeit. (Skala von 1-stark verrringert bis 5-stark erhöht)
(4) Und wie hat sich Ihre Paarbeziehung durch die Corona-Krise verändert? Die Konflikte in meiner Familie/Beziehung. (Skala von 1-stark verrringert bis 5-stark erhöht)
(5) Vor der Corona-Krise: Verbringen Sie Zeit damit, chronisch kranke, behinderte oder alte und pflegebedürftige Familienmitglieder, Freunde oder Nachbarn zu betreuen oder ihnen zu helfen? (Angabe in Stunden)
(6) Derzeit: Verbringen Sie Zeit damit, chronisch kranke, behinderte oder alte und pflegebedürftige Familienmitglieder, Freunde oder Nachbarn zu betreuen oder ihnen zu helfen? (Angabe in Stunden)

Themenbereich: Corona-Folgen	Kinder-betreuung	Arbeitsstunden	Zeit-, Erfolgsdruck	Home Office	Anerkennung	Autonomie	Sicherheit Job	Planung Arbeit	Überwachung im Job	Persönlicher Kontakt im Job	Vereinbarkeit	Infektions-risiko am Arbeitsplatz
	(1)+	(2)+	(3)+	(4)+	(5)+	(6)+	(7)+	(8)+	(9)+	(10)+	(11)++	(12)++
Gender (Ref.: Männer)												
Frauen	-0.01	0.01	0.01	0.00	0.01	-0.02	0.03*	0.01	0.01	0.01	0.05	0.03
Alter (Ref.: 20-29)												
30-39	-0.03	0.01	0.01	0.06**	-0.02	0.01	0.01	0.01	0.02	-0.02	0.14***	-0.02
40-49	0.00	-0.00	0.02	0.07**	-0.01	0.00	0.03	-0.01	0.01	-0.05*	0.11**	-0.11**
50-59	0.09	-0.00	0.02	0.08***	-0.00	0.00	0.01	-0.00	0.02	-0.06**	0.01	-0.09*
60-64	0.56*	0.01	-0.02	0.08	0.03	-0.01	0.01	-0.01	0.02	-0.01	-0.13	-0.07
Bildung (Ref.: Pflichtschule)												
Lehre	-0.05	-0.06*	0.03	0.03	-0.02	-0.01	0.02	-0.03	-0.00	0.01	0.07	0.09
BMS	0.01	-0.08*	0.00	0.05	-0.02	0.01	0.03	-0.02	-0.01	-0.02	0.04	0.16*
AHS-Matura	-0.09	-0.00	0.01	0.09	0.02	-0.00	0.04	-0.00	0.01	-0.02	0.08	0.04
BHS-Matura	0.00	-0.03	0.02	0.11**	-0.01	-0.01	0.00	0.01	-0.01	-0.03	0.05	0.03
Diplom, Univ.-Lehrgang	-0.08	-0.04	-0.02	0.13**	0.04	0.00	0.01	-0.00	-0.01	-0.04	0.12	0.11
Hochschule	-0.13*	-0.04	0.03	0.19***	-0.01	-0.01	-0.04	-0.00	-0.02	-0.10**	0.14*	0.02
Bundesland (Ref.: Vorarlberg)												
Tirol	0.02	-0.07*	-0.01	0.05	0.03	-0.01	0.01	-0.01	0.01	-0.10**	0.01	0.04
Salzburg	0.07	-0.02	-0.01	0.09	0.00	0.01	-0.02	-0.01	-0.05	-0.09*	-0.01	-0.02
Oberösterreich	0.06	-0.05	0.01	0.08*	0.01	-0.00	-0.03	-0.03	-0.01	-0.10**	-0.05	-0.08
Kärnten	0.18*	-0.04	-0.01	0.05	0.05	-0.00	0.02	0.00	-0.02	-0.04	-0.15	0.01
Steiermark	0.07	-0.03	0.02	0.08*	0.03	0.00	-0.00	0.03	-0.00	-0.10**	0.02	0.00
Burgenland	0.02	-0.00	0.02	0.03	0.01	-0.01	0.01	-0.01	-0.01	-0.10*	-0.01	0.01
Niederösterreich	0.11	-0.06*	0.01	0.10**	-0.00	-0.01	-0.03	-0.00	-0.02	-0.11**	-0.06	0.00
Wien	-0.02	-0.07*	-0.03	0.12**	-0.01	-0.02	-0.02	-0.01	-0.02	-0.15***	-0.03	-0.01
Wohnort (Ref.: Land)												
Größeres Dorf/ Kleinstadt	-0.06	-0.03*	-0.02	-0.00	-0.00	-0.01	-0.04*	-0.01	-0.01	-0.01	0.01	0.01
Mittelstadt	-0.06	0.01	0.01	-0.00	-0.02	-0.00	-0.04	-0.01	0.01	-0.01	-0.00	0.02
Großstadt/ Vorstadt	0.00	0.01	0.03	0.02	-0.00	-0.00	-0.01	0.00	-0.01	0.01	0.03	0.06
Staatsbürgerschaft												
AUT	0.01	0.03	0.07**	0.04	0.03	-0.02	0.03	0.05**	-0.00	-0.04	-0.01	-0.13*
Tätigkeit (Ref.: Vollzeit)												
Teilzeit	-0.01	-0.04**	-0.01	-0.04	0.02	-0.00	0.01	-0.02	0.00	0.00	0.08*	0.01
Kurzarbeit	0.02	-0.28***	-0.05**	0.00	-0.05**	-0.05***	-0.06***	-0.03*	0.01	-0.07***	-0.02	0.00
Ausbildung	-0.21											
Arbeitslos	-0.01											
Karenz, Hausarbeit	0.14**	-0.12***	-0.07	-0.10*	-0.01	-0.01	-0.12**	-0.06*	-0.05	-0.03	0.06	0.01
Pension, Arbeitsunfähig	-0.07											
Mode (Ref.: CATI)												
CAWI	-0.04	-0.03	-0.03	0.01	-0.03	-0.02	-0.05**	-0.00	0.00	0.00	-0.02	0.02
_cons	0.61***	0.59***	0.44***	0.33***	0.52***	0.55***	0.51***	0.47***	0.51***	0.55***	0.21*	0.34***
N	798	1357	1348	1162	1297	1323	1359	1351	1338	1360	1421	1448
R-sq	0.070	0.231	0.040	0.104	0.043	0.026	0.051	0.037	0.018	0.070	0.050	0.033

Quelle: ANONYM-Survey, Daten erhoben im Juni 2020. Sample: in Österreich lebende Personen im Alter von 20-64.

+ Lineare Regressionsmodelle; die abhängigen Variablen wurden auf den Wertebereich 0 bis 1 standardisiert.

++ Lineares Wahrscheinlichkeitsmodell; die abhängige Variable wurde dichotomisiert.

**** p<0.001; ** p<0.01; * p<0.05*

(1) Die **Kinderbetreuung** stellt seit Beginn der Corona-Krise ein Problem für mich dar. (Skala von 1-stimme voll und ganz zu bis 5-stimme überhaupt nicht zu)
(2) Wie hat sich Ihre berufliche Situation seit Beginn der Corona-Krise geändert? **Die Anzahl der Stunden, die ich pro Woche arbeite**. (Skala von 1-stark verringert bis 5-stark erhöht)
(3) Wie hat sich Ihre berufliche Situation seit Beginn der Corona-Krise geändert? **Der Zeit- oder Erfolgsdruck in der Arbeit**. (Skala von 1-stark verringert bis 5-stark erhöht)
(4) Wie hat sich Ihre berufliche Situation seit Beginn der Corona-Krise geändert? **Wie viel ich von zu Hause aus arbeite** (Skala von 1-stark verringert bis 5-stark erhöht)
(5) Wie hat sich Ihre berufliche Situation seit Beginn der Corona-Krise geändert? **Die gesellschaftliche Anerkennung für meine Tätigkeit** (Skala von 1-stark verringert bis 5-stark erhöht)
(6) Wie hat sich Ihre berufliche Situation seit Beginn der Corona-Krise geändert? **Wie sehr ich Entscheidungen, die wichtig für meine Arbeit sind, beeinflussen kann**. (Skala von 1-stark verringert bis 5-stark erhöht)
(7) Wie hat sich Ihre berufliche Situation seit Beginn der Corona-Krise geändert? **Die Sicherheit meines Arbeitsplatzes** (Skala von 1-stark verringert bis 5-stark erhöht)
(8) Wie hat sich Ihre berufliche Situation seit Beginn der Corona-Krise geändert? **Wie sehr ich meine Arbeit selbst planen/einteilen kann** (Skala von 1-stark verringert bis 5-stark erhöht)
(9) Wie hat sich Ihre berufliche Situation seit Beginn der Corona-Krise geändert? **Wie stark meine Arbeit überwacht wird** (Skala von 1-stark verringert bis 5-stark erhöht)
(10) Wie hat sich Ihre berufliche Situation seit Beginn der Corona-Krise geändert? **Der persönliche Kontakt zu Menschen** (Kollegen(innen), Vorgesetzte, Kunden(innen), Klient(innen), Patienten(innen), usw.) (Skala von 1-stark verringert bis 5-stark erhöht)
(11) Würden Sie sagen, dass es seit Beginn der Corona-Krise einfacher oder schwieriger geworden ist, **Beruf und Familie miteinander zu vereinbaren**? (Skala 1-einfacher, 2-gleich, 3-schwieriger, dichotomisiert in 1=schwieriger)
(Ein ordered logit Modell mit drei Kategorien führt zu vergleichbaren Ergebnissen)
(12) Wie hoch schätzen Sie das **Risiko ein, dass Sie sich im Rahmen Ihrer beruflichen Tätigkeit mit dem Corona-Virus anstecken**? (Skala 1-sehr hoch bis 4-sehr niedrig, dichotomisiert in 1=hoch)
(Ein ordered logit Modell mit drei Kategorien führt zu vergleichbaren Ergebnissen)

Themenbereich: Arbeitsbeziehungen	Betriebsrat vorhanden	Home Office	Arbeitsstunden	Wissen Vorgesestzte	Autonomie im Job	Tätigkeit Probleme lösen	Tätigkeit eintönig	Tätigkeit Dinge lernen	Tätigkeit eigene Ideen
	(1)++	(2)+	(3)+	(4)+	(5)+	(6)+	(7)+	(8)+	(9)+
Gender (Ref.: Männer)									
Frauen	-0.13***	0.01	-0.06***	-0.01	-0.00	0.06***	0.01	0.06***	0.05**
Alter (Ref.: 20-29)									
30-39	0.05	-0.02	-0.01	-0.04	0.07***	-0.04	0.05*	0.01	0.02
40-49	0.13**	-0.07*	0.01	-0.07**	0.11***	-0.02	0.10***	0.02	0.03
50-59	0.11*	-0.10***	-0.00	-0.07**	0.14***	-0.02	0.12***	0.02	0.02
60-64	-0.00	-0.08	-0.01	-0.06	0.14**	-0.13**	0.18***	-0.07	-0.12*
Bildung (Ref.: Pflichtschule)									
Lehre	0.00	0.03	-0.02	-0.03	0.05	-0.01	0.09*	-0.02	0.01
BMS	0.04	-0.01	-0.02	-0.05	0.08*	-0.07*	0.08*	-0.07*	-0.04
AHS-Matura	0.05	-0.11	-0.03	-0.06	0.06	0.01	0.10*	-0.09*	-0.03
BHS-Matura	0.09	-0.14**	-0.02	-0.08	0.09*	-0.05	0.12**	-0.05	-0.00
Diplom, Univ.-Lehrgang	0.15	-0.07	-0.03	-0.02	0.04	-0.10*	0.19***	-0.15***	-0.08
Hochschule	0.02	-0.24***	-0.03	-0.03	0.15***	-0.09*	0.13***	-0.15***	-0.10**
Bundesland (Ref.: Vorarlberg)									
Tirol	-0.02	-0.06	0.01	0.00	0.05	-0.15***	-0.01	-0.01	-0.01
Salzburg	0.00	-0.06	0.00	0.02	0.03	-0.07	-0.02	-0.01	0.00
Oberösterreich	0.06	-0.09	0.01	-0.00	0.04	-0.13***	-0.00	-0.04	-0.02
Kärnten	-0.02	-0.03	0.02	-0.02	0.02	-0.14***	-0.01	-0.03	-0.04
Steiermark	0.08	-0.10*	0.03	0.02	0.02	-0.13***	0.00	-0.02	-0.01
Burgenland	0.08	-0.03	0.04	0.08	-0.05	-0.03	-0.01	-0.05	0.05
Niederösterreich	0.10	-0.12*	0.02	0.01	0.01	-0.12***	-0.02	-0.02	0.01
Wien	0.14	-0.17**	0.01	-0.02	0.01	-0.18***	-0.03	-0.07	-0.07
Wohnort (Ref.: Land)									
Größeres Dorf/ Kleinstadt	-0.04	-0.03	-0.01	0.02	-0.01	-0.03	-0.03	-0.03	-0.01
Mittelstadt	-0.01	-0.01	0.01	0.00	-0.02	0.02	-0.02	-0.00	0.03
Großstadt/ Vorstadt	-0.02	0.00	0.01	0.02	-0.02	0.05*	-0.04	0.05*	0.06*
Staatsbürgerschaft									
AUT	0.03	0.08*	-0.01	-0.09**	0.04	0.00	0.09**	0.04	0.04
Tätigkeit (Ref.: Vollzeit)									
Teilzeit	-0.02	0.07**	-0.19***	-0.07**	-0.01	0.03	-0.01	-0.01	0.03
Kurzarbeit	-0.09*	-0.02	-0.20***	0.02	-0.03	-0.01	-0.03	0.03	0.02
Karenz, Hausarbeit	-0.11	0.05	-0.36***	0.02	-0.05	0.07	-0.03	0.06	-0.00
Mode (Ref.: CATI)									
CAWI	0.01	-0.01	-0.01	0.04	0.00	-0.01	-0.04	0.02	0.01
_cons	0.50***	0.85***	0.55***	0.51***	0.42***	0.47***	0.37***	0.44***	0.43***
N	1314	1522	1290	1307	1379	1392	1392	1392	1392
R-sq	0.061	0.106	0.493	0.044	0.068	0.076	0.079	0.070	0.053
Quelle: ANONYM-Survey, Daten erhoben im Juni 2020. Sample: in Österreich lebende Personen im Alter von 20-64.									

+ Lineare Regressionsmodelle; die abhängigen Variablen wurden auf den Wertebereich 0 bis 1 standardisiert.
++ Lineares Wahrscheinlichkeitsmodell; die abhängige Variable wurde dichotomisiert.
**** p<0.001; ** p<0.01; * p<0.05*

(1) Gibt es in Ihrem Betrieb bzw. in der Organisation, für die Sie arbeiten, einen Betriebsrat? (0-nein, 1-ja)
(2) Wie oft arbeiten Sie derzeit von zu Hause aus? (von 1-täglich bis 5-nie)
(3) Wie viele Stunden arbeiten Sie derzeit durchschnittlich pro Woche? (Angabe in Stunden)
(4) Wie einfach oder schwierig ist es für Ihre direkten Vorgesetzten zu wissen, wie groß Ihr Einsatz bei der Arbeit ist? (Skala von 0-äußerst einfach bis 10-äußerst schwierig)
(5) Skala: Summenindex aus vier Indikatoren zu Arbeitsautonomie (von 1-keine Selbstbestimmung bis 5-starke Selbstbestimmung)
(6) Wie häufig schließt Ihre derzeitige berufliche Tätigkeit Folgendes ein? das selbständige Lösen unvorhergesehener Probleme (Skala von 1-immer bis 6-nie)
(7) Wie häufig schließt Ihre derzeitige berufliche Tätigkeit Folgendes ein? eintönige Aufgaben (Skala von 1-immer bis 6-nie)
(8) Wie häufig schließt Ihre derzeitige berufliche Tätigkeit Folgendes ein? das Lernen neuer Dinge (Skala von 1-immer bis 6-nie)
(9) Wie häufig schließt Ihre derzeitige berufliche Tätigkeit Folgendes ein? das Umsetzen meiner eigenen Ideen (Skala von 1-immer bis 6-nie)
